# Supplementary material for: Autocrine INSL5 promotes tumor progression and glycolysis via activation of STAT5 signaling
Source: EMBO Mol Med. 2020 Jul 12;12(9):e12050. doi: 10.15252/emmm.202012050 (PMC7507000; doi:10.15252/emmm.202012050)
Supplement: Supplementary file 1 — Appendix [file EMMM-12-e12050-s001.doc]

**Autocrine INSL5 promotes tumor progression and glycolysis via activation of STAT5 signaling**

Shi-Bing Li^1,11^, Yan-Yan Liu^2,11^, Li Yuan^1,11^, Ming-Fang Ji^3,11^, Ao Zhang^1^, Hui-Yu Li^4^, Lin-Quan Tang^1,8^, Shuo-Gui Fang^1,9^, Hua Zhang^10^, Shan Xing^1^, Man-Zhi Li^1^, Qian Zhong^1^, Shao-Jun Lin^5^, Wan-Li Liu^1^, Peng Huang^1^, Yi-Xin Zeng^1^, Yu-Ming Zheng^6, *^, Zhi-Qiang Ling^7, *^, Jian-Hua Sui^4, *^ and Mu-Sheng Zeng^1,12,*^

**APPENDIX.**

**Contents: Appendix Tables S1 – S4**

**Appendix Table S1 – Page 2**

Characteristics of the patients.

**Appendix Table S2 – Page 3**

Univariate analysis and Multivariate analysis with the COX proportional hazards for the predictor of OS and DFS of the NPC patients in GZ

**Appendix Table S3 – Page 4**

Primer sequences for qRT-PCR

**Appendix Table S4 – Page 5**

Summary of exact *P*-values in figures

**Appendix Table S1**: Characteristics of the patients.

|  | GZ cohort（n=303） | | | | ZJ cohort（n=159） | | | | ZS cohort（n=197） | | | |
| --- | --- | --- | --- | --- | --- | --- | --- | --- | --- | --- | --- | --- |
| Characteristics | Overall | Low INSL5 | High INSL5 | P  Value | Overall | Low INSL5 | High INSL5 | P  Value | Overall | Low INSL5 | High INSL5 | P  Value |
| Patients | 303 | 117 | 186 |  | 159 | 133 | 26 |  | 197 | 186 | 11 |  |
| Age |  |  |  |  |  |  |  |  |  |  |  |  |
| Median | 47 |  |  | - | 56 |  |  | - | 47 |  |  | - |
| Range | 13-76 |  |  |  | 19-82 |  |  |  | 17-81 |  |  |  |
| Gender |  |  |  |  |  |  |  |  |  |  |  |  |
| Male | 64 | 26 | 38 | 0.773 | 47 | 40 | 7 | 0.747 | 51 | 50 | 1 | 0.191 |
| Female | 239 | 91 | 148 |  | 112 | 93 | 19 |  | 146 | 136 | 10 |  |
| BMI |  |  |  |  |  |  |  |  |  |  |  |  |
| ≤21.4 | 84 | 39 | 45 | 0.090 | - | - | - | - | - | - | - |  |
| ＞21.4 | 219 | 78 | 141 |  | - | - | - |  | - | - | - |  |
| T stage |  |  |  |  |  |  |  |  |  |  |  |  |
| T1-T2 | 30 | 17 | 13 | **0.047** | 18 | 14 | 4 | 0.475 | 54 | 48 | 6 | 0.143 |
| T3-T4 | 273 | 100 | 173 |  | 141 | 119 | 22 |  | 87 | 83 | 4 |  |
| N stage |  |  |  |  |  |  |  |  |  |  |  |  |
| N0 | 34 | 16 | 18 | 0.350 | 6 | 5 | 1 | 0.983 | 11 | 11 | 0 | 0.340 |
| N1-N3 | 269 | 101 | 168 |  | 153 | 128 | 25 |  | 130 | 120 | 10 |  |
| EBV DNA |  |  |  |  |  |  |  |  |  |  |  |  |
| Low DNA | 181 | 73 | 108 | 0.473 | 1 | 1 | 0 | 0.657 | 131 | 126 | 5 | 0.128 |
| High DNA | 122 | 44 | 78 |  | 158 | 132 | 26 |  | 66 | 60 | 6 |  |
| LDH(u/L) |  |  |  |  |  |  |  |  |  |  |  |  |
| ≤245 | 281 | 108 | 173 | 0.823 |  | - | - | - | - | - | - | - |
| ＞245 | 22 | 9 | 13 |  |  | - | - |  | - | - | - |  |
| hs-CRP(g/ml) |  |  |  |  |  |  |  |  |  |  |  |  |
| ≤3 | 186 | 76 | 110 | 0.334 | - | - | - | - | - | - | - | - |
| ＞3 | 117 | 41 | 76 |  | - | - | - |  | - | - | - |  |
| VCA-IgA |  |  |  |  |  |  |  |  |  |  |  |  |
| ＜1:160 | 89 | 39 | 50 | 0.245 | 134 | 115 | 19 | 0.086 | - | - | - | - |
| ≥1:160 | 214 | 78 | 136 |  | 25 | 18 | 7 |  | - | - | - |  |
| EA-IgA |  |  |  |  |  |  |  |  |  |  |  |  |
| ＜1:20 | 121 | 53 | 68 | 0.149 | 2 | 2 | 0 | 0.529 | - | - | - | - |
| ≥1：20 | 182 | 64 | 118 |  | 157 | 131 | 26 |  | - | - | - |  |
| Progression |  |  |  |  |  |  |  |  |  |  |  |  |
| Yes | 60 | 16 | 44 | **0.038** | - | - | - | - | - | - | - | - |
| No | 243 | 101 | 142 |  | - | - | - |  | - | - | - |  |
| Death |  |  |  |  |  |  |  |  |  |  |  |  |
| Yes | 41 | 8 | 33 | **0.009** | - | - | - | - | - | - | - | - |
| NO | 262 | 109 | 153 |  | - | - | - |  | - | - | - |  |

^a^Chi-square test

**Appendix Table S2**: Univariate analysis and Multivariate analysis with the COX proportional hazards for the predictor of OS and DFS of the NPC patients in GZ cohort

|  | Univariate analysis | | |  | Multivariate analysis | | |
| --- | --- | --- | --- | --- | --- | --- | --- |
| Prognostic factors | HR | 95% CI | *P* | HR | | 95% CI | P |
| OS |  |  |  |  | |  |  |
| Age (years) | 1.654 | 0.885-3.090 | 0.115 | 1.025 | | 0.996-1.054 | 0.095 |
| Gender male | 0.941 | 0.449-1.971 | 0.871 |  | |  |  |
| T stage | 2.379 | 0.574-9.858 | 0.232 | 2.175 | | 0.512-9.247 | 0.292 |
| N stage | 2.692 | 0.650-11.152 | 0.172 | 2.676 | | 0.640-11.186 | 0.177 |
| VCA-IgA | 0.658 | 0.348-1.243 | 0.197 |  | |  |  |
| EA-IgA | 0.988 | 0.527-1.854 | 0.971 |  | |  |  |
| EBV DNA | 1.898 | 1.027-3.509 | **0.041** | 1.636 | | 0.862 | 3.103 |
| BMI | 2.720 | 1.067-6.935 | **0.036** | 2.663 | | 1.034-6.853 | **0.042** |
| LDH | 0.629 | 0.152-2.608 | 0.523 |  | |  |  |
| CRP | 2.136 | 1.152-3.961 | **0.016** | 1.764 | | 0.928-3.352 | 0.083 |
| INSL5 | 2.905 | 1.341-6.292 | **0.007** | 2.337 | | 1.070-5.103 | **0.033** |
| DFS |  |  |  |  | |  |  |
| Age (years) | 1.498 | 0.898-2.499 | 0.122 | 1.513 | | 0.905-2.531 | 0.114 |
| Gender | 0.712 | 0.402-1.261 | 0.244 |  | |  |  |
| T stage | 1.385 | 0.553-3.470 | 0.487 |  | |  |  |
| N stage | 1.952 | 0.708-5.384 | 0.196 |  | |  |  |
| VCA-IgA | 1.754 | 0.990-3.110 | **0.054** | 1.466 | | 0.818-2.629 | 0.199 |
| EA-IgA | 1.304 | 0.762-2.231 | 0.334 |  | |  |  |
| EBV DNA | 2.081 | 1.251-3.463 | **0.005** | 1.880 | | 1.114-3.171 | **0.018** |
| BMI | 1.243 | 0.683-2.261 | 0.476 |  | |  |  |
| LDH | 2.532 | 1.245-5.148 | **0.010** | 1.874 | | 0.897-3.916 | 0.095 |
| CRP | 1.508 | 0.908-2.506 | 0.113 |  | |  |  |
| INSL5 | 1.883 | 1.063-3.338 | **0.030** | 1.794 | | 1.008-3.193 | **0.047** |

Age, ≥47 vs ＜47(OS), ＞50 vs ≤50(DFS); Gender, male vs female; T stage, T1-T2 vs T3-T4; N stage, N0-N1 vs N2-N3; VCA-IgA, ≥1:160 vs <1:160(OS), ＞320 vs ≤320(DFS) ; EA-IgA, ≥1:20 vs <1:20; EBV DNA(copy/ml), >4000 vs ≤4000; INSL5(ng/ml), ≥3.73 vs ＜3.73; BMI(kg/m^2^), ＞21.4 vs ≤21.4；LDH(u/L), ＞245 vs ≤245; hs-CRP(g/ml), ≥3 vs ＜3.

**Appendix Table S3**: Primer sequences for qRT-PCR

| Target | Sense (5’-3’) | Antisense (5’-3’) |
| --- | --- | --- |
| INSL5 | TGTGAGACTCTGTGGGCTAGA | TGGGAGCTGGAAGGAGTTTC |
| GPCR142 | CTGATGATGCTCCGATGCCT | TGAAAGTCCAGTGCCGACTC |
| HK2 | GATTGCCTCGCATCTGCTTG | GCTCCAAGCCCTTTCTCCAT |
| Glut3 | GTCATGATCCCAGCGAGACC | CTGGGGTGACCTTCTGTGTC |
| PFK1 | CAGGCTCCCTCCATCCTCA | GGAGTCCTGACTAACTGGCG |
| Enolase 1 | CGGGAATCCCACTGTTGAGG | TTCTTGCTAACCAGGGCAGG |
| HK2-DNA | CACTGTGCCTGGCCTATAAC | CCTCTGTGGTCCCATTTCA |
| Glut3-DNA | TGGACAGCACTGCCCTTTG | TGATTTGATGACAACTCCCCTC |
| PFK1-DNA | GCTGACCGGGTCCGACACAGT | TCTTTGACCTCCTGCCCCAGC |
| β-actin | GTGAAGGTGACAGCAGTCGGT | AAGTGGGGTGGCTTTTAGGAT |
| GAPDH-DNA | CCCCACACACATGCACTTACC | CCTAGTCCCAGGGCTTTGATT |

**Appendix Table S4**. Summary of exact *P*-values in figures

| Figure | | | Exact *P*-value | |  |
| --- | --- | --- | --- | --- | --- |
| Fig 1 | | B | NPEC vs. NPC: *P*=0.039 | |  |
|  |  | C | Normal (EBV-) vs. Normal (EBV+): P=0.0259; Normal (EBV+) vs. NPC: P<0.0001; Normal (EBV-) vs. NPC: P<0.0001 | |  |
|  |  | E | Normal (EBV-) vs. Normal (EBV+): P<0.0001; Normal (EBV+) vs. NPC: P<0.0001; Normal (EBV-) vs. NPC: P<0.0001 | |  |
|  |  | F | Normal (EBV-) vs. Normal (EBV+): P=0.0027; Normal (EBV+) vs. NPC: P<0.0001; Normal (EBV-) vs. NPC: P<0.0001 | |  |
| Fig 3 | | B | CNE1-vector vs. CNE1-INSL5: P=0.0169; CNE1-INSL5-NC vs CNE1-INSL5-siGPCR142 #1: P=0.013; CNE1-INSL5-NC vs CNE1-INSL5-siGPCR142 #2: P=0.0007;  CNE2-vector vs. CNE2-INSL5: P=0.0314; CNE2-INSL5-NC vs CNE2-INSL5-siGPCR142 #1: P<0.0001; CNE2-INSL5-NC vs CNE2-INSL5-siGPCR142 #2: P<0.0001;  HK1-vector vs. HK1-INSL5: P<0.0001; HK1-INSL5-NC vs HK1-INSL5-siGPCR142 #1: P<0.0001; HK1-INSL5-NC vs HK1-INSL5-siGPCR142 #2: P=0.0178; | |  |
|  |  | D | CNE1-vector vs. CNE1-INSL5: P<0.0001; CNE1-INSL5-NC vs CNE1-INSL5-siGPCR142 #1: P=0.0001; CNE1-INSL5-NC vs CNE1-INSL5-siGPCR142 #2: P=0.0003;  CNE2-vector vs. CNE2-INSL5: P<0.0001; CNE2-INSL5-NC vs CNE2-INSL5-siGPCR142 #1: P=0.0056; CNE2-INSL5-NC vs CNE2-INSL5-siGPCR142 #2: P=0.0006;  HK1-vector vs. HK1-INSL5: P=0.0029; HK1-INSL5-NC vs HK1-INSL5-siGPCR142 #1: P=0.0043; HK1-INSL5-NC vs HK1-INSL5-siGPCR142 #2: P=0.0239; | |  |
|  |  | F | CNE1-vector vs. CNE1-INSL5: P=0.0002; CNE1-INSL5-NC vs CNE1-INSL5-siGPCR142 #1: P=0.0008; CNE1-INSL5-NC vs CNE1-INSL5-siGPCR142 #2: P=0.0004;  CNE2-vector vs. CNE2-INSL5: P<0.0001; CNE2-INSL5-NC vs CNE2-INSL5-siGPCR142 #1: P=0.0061; CNE2-INSL5-NC vs CNE2-INSL5-siGPCR142 #2: P=0.0025;  HK1-vector vs. HK1-INSL5: P=0.0001; HK1-INSL5-NC vs HK1-INSL5-siGPCR142 #1: P=0.0041; HK1-INSL5-NC vs HK1-INSL5-siGPCR142 #2: P=0.0009; | |  |
|  |  | H | CNE1-vector vs. CNE1-INSL5: P<0.0001; CNE1-INSL5-NC vs CNE1-INSL5-siGPCR142 #1: P=0.0012; CNE1-INSL5-NC vs CNE1-INSL5-siGPCR142 #2: P<0.0001;  CNE2-vector vs. CNE2-INSL5: P=0.0001; CNE2-INSL5-NC vs CNE2-INSL5-siGPCR142 #1: P=0.0006; CNE2-INSL5-NC vs CNE2-INSL5-siGPCR142 #2: P=0.0002;  HK1-vector vs. HK1-INSL5: P<0.0001; HK1-INSL5-NC vs HK1-INSL5-siGPCR142 #1: P=0.0015; HK1-INSL5-NC vs HK1-INSL5-siGPCR142 #2: P=0.0003; | |  |
|  |  | I | Day 11, HK1-vector vs.HK1-INSL5: P=0.033; Day 12, HK1-vector vs.HK1-INSL5: P=0.0437; Day 13, HK1-vector vs.HK1-INSL5: P=0.0351; Day 14, HK1-vector vs.HK1-INSL5: P=0.0109 | |  |
|  |  | K | HK1-vector vs. HK1-INSL5: P=0.0005 | |  |
| Fig 4 | | G | CNE1-vector vs. CNE1-INSL5: P=0.0058; CNE2-vector vs. CNE2-INSL5: P=0.0161; HK1-vector vs. HK1-INSL5: P=0.0053 | |  |
|  | | H | CNE1-vector vs. CNE1-INSL5: P=0.0067 | |  |
|  | | I | CNE1-vector vs. CNE1-INSL5, Glu: P=0.0345; CNE1-vector vs. CNE1-INSL5, Lac: P=0.0229 | |  |
|  | | J | CNE1-vector vs. CNE1-INSL5: P=0.0399; CNE1-vector Oligomycin vs. CNE1-INSL5 Oligomycin: P=0.0438  CNE1-vector 2-DGvs. CNE1-INSL5 2-DG: P=0.0168 | |  |
|  | | K | CNE1-vector vs. CNE1-INSL5: P=0.0088 | |  |
|  | | L | HNE1-EBV siNC vs. HNE1-EBV siINSL5 1#, ATP: P=0.0349  HNE1-EBV siNC vs. HNE1-EBV siINSL5 2#, ATP: P=0.0344  HNE1-EBV siNC vs. HNE1-EBV siINSL5 1#, HK2: P=0.0043  HNE1-EBV siNC vs. HNE1-EBV siINSL5 1#, HK2: P=0.0049  HNE1-EBV siNC vs. HNE1-EBV siINSL5 1#, Lac: P=0.0125  HNE1-EBV siNC vs. HNE1-EBV siINSL5 1#, Lac: P=0.003 | |  |
| Fig 5 | | F | HK2-vector vs. HK2-STAT5: P=0.044; Glut3-vector vs. Glut3-STAT5: P=0.0473; PFK1-vector vs. PFK1-STAT5: P=0.0007 | |  |
|  | | G | HK2-vector vs. HK2-STAT5: P=0.0274; Glut3-vector vs. Glut3-STAT5: P=0.0024; PFK1-vector vs. PFK1-STAT5: P=0.0022 | |  |
|  | | H | CNE1-vector vs. CNE1-INSL5: P=0.0421;  CNE1-INSL5-siNC vs CNE1-INSL5-siSTAT5 1#: P=0.0328;  CNE1-INSL5-siNC vs CNE1-INSL5-siSTAT5 2#: P=0.0368 | |  |
|  | | I | HK1-vector vs. HK1-INSL5: P=0.0017;  HK1-INSL5-DMSO vs. HK1-INSL5-Ruxo: P=0.0079 | |  |
|  | | J | HK1-vector vs. HK1-INSL5: P=0.0116;  HK1-INSL5-siNCvs.HK1-INSL5-siSTAT5 1#: P=0.0025;  HK1-INSL5-siNC vs.HK1-INSL5-siSTAT5 2#: P=0.0008  HK1-INSL5-DMSO vs. HK1-INSL5-Ruxo: P<0.0001 | |  |
| Fig 6 | | A | rINSL5(-) vs. rINSL5 (+): P<0.0001  rINSL5+IgG vs. rINSL5+αINSL5-50：P=0.0042  rINSL5+IgG vs. rINSL5+αINSL5-50：P=0.0002 | |  |
|  | | B | rINSL5(-) vs. rINSL5 (+), migration: P=0.006  rINSL5+IgG vs. rINSL5+αINSL5-50, migration：P=0.0004  rINSL5+IgG vs. rINSL5+αINSL5-50, migration：P=0.0001  rINSL5(-) vs. rINSL5 (+), invasion: P=0.0003  rINSL5+IgG vs. rINSL5+αINSL5-50, invasion：P=0.0006  rINSL5+IgG vs. rINSL5+αINSL5-50, invasion：P=0.0002 | |  |
|  | | G | IgG vs. αINSL5: P=0.0116; IgG vs. αGPCR142: P=0.0102 | |  |
|  | | H | IgG vs. αINSL5: P=0.0299; IgG vs. αGPCR142: P=0.026 | |  |
| Fig EV1 | | B | CNE2-EBV (-) vs. CNE2-EBV (+): P=0.0425  HNE1-EBV (-) vs. HNE1-EBV (+): P=0.3713 | |  |
|  |  | C | NPC vs. NPC: P<0.0001 | |  |
| Fig EV2 | | C | NP69-vector vs. NP69-INSL5: P<0.0001 | |  |
|  | | D | NP69-vector vs. NP69-INSL5: P=0.0006 | |  |
|  | | E | NP69-vector vs. NP69-INSL5: P=0.001 | |  |
|  | | F | NP69-vector vs. NP69-INSL5, migration: P=0.0001  NP69-vector vs. NP69-INSL5, invasion: P=0.0003 | |  |
|  | | G | CNE1-vector vs. CNE1-INSL5: P<0.0001; CNE1-INSL5-NC vs CNE1-INSL5-siGPCR142 #1: P=0.0013; CNE1-INSL5-NC vs CNE1-INSL5-siGPCR142 #2: P=0.0005;  CNE2-vector vs. CNE2-INSL5: P<0.0001; CNE2-INSL5-NC vs CNE2-INSL5-siGPCR142 #1: P=0.0164; CNE2-INSL5-NC vs CNE2-INSL5-siGPCR142 #2: P=0.0019;  HK1-vector vs. HK1-INSL5: P<0.0001; HK1-INSL5-NC vs HK1-INSL5-siGPCR142 #1: P=0.0003; HK1-INSL5-NC vs HK1-INSL5-siGPCR142 #2: P=0.0002; | |  |
|  | | H | CNE2-EBV-siNC vs. CNE2-EBV-siINSL5 1#: P=0.0112;  CNE2-EBV-siNC vs. CNE2-EBV-siINSL5 2#: P=0.0205;  HNE1-EBV-siNC vs. HNE1-EBV-siINSL5 1#: P<0.0001;  HNE1-EBV-siNC vs. HNE1-EBV-siINSL5 2#: P<0.0001; | |  |
|  | | I | CNE2-EBV-siNC vs. CNE2-EBV-siINSL5 1#, migration: P<0.0001; CNE2-EBV-siNC vs. CNE2-EBV-siINSL5 2#, migration: P=0.0001;  CNE2-EBV-siNC vs. CNE2-EBV-siINSL5 1#, invasion: P=0.0003; CNE2-EBV-siNC vs. CNE2-EBV-siINSL5 2#, invasion: P=0.001;  HNE1-EBV-siNC vs. HNE1-EBV-siINSL5 1#, migration: P=0.0009; HNE1-EBV-siNC vs. HNE1-EBV-siINSL5 2#, migration: P=0.0009;  HNE1-EBV-siNC vs. HNE1-EBV-siINSL5 1#, invasion: P=0.0091; HNE1-EBV-siNC vs. HNE1-EBV-siINSL5 2#, invasion: P=0.0015; | |  |
|  | | J | CNE2-EBV-siNC vs. CNE2-EBV-siINSL5 1#: P=0.0249;  CNE2-EBV-siNC vs. CNE2-EBV-siINSL5 2#: P=0.0006;  HNE1-EBV-siNC vs. HNE1-EBV-siINSL5 1#: P=0.0464;  HNE1-EBV-siNC vs. HNE1-EBV-siINSL5 2#: P=0.0235; | |  |
|  | | K | CNE2-vector vs. CNE2-INSL5, day 18: P=0.0368;  CNE2-vector vs. CNE2-INSL5, day 20: P=0.0249 | |  |
|  | | L | CNE2-vector vs. CNE2-INSL5: P=0.0061 | |  |
| Fig EV3 | | C | CNE1-vector vs. CNE1-INSL5, F1,6BP: P=0.0032;  CNE1-vector vs. CNE1-INSL5, DHAP: P=0.0006  CNE1-vector vs. CNE1-INSL5, F-6-P: P=0.448  CNE1-vector vs. CNE1-INSL5, G3P: P=0.0328  CNE1-vector vs. CNE1-INSL5, PEP: P=0.0002  CNE1-vector vs. CNE1-INSL5, 3-PG: P=0.001 | |  |
|  | | D | CNE2-vector vs. CNE2-INSL5, F1,6BP: P=0.0002;  CNE2-vector vs. CNE2-INSL5, DHAP: P<0.0001  CNE2-vector vs. CNE2-INSL5, F-6-P: P=0.0002  CNE2-vector vs. CNE2-INSL5, G3P: P=0.1604  CNE2-vector vs. CNE2-INSL5, PEP: P=0.0446  CNE2-vector vs. CNE2-INSL5, 3-PG: P=0.1478 | |  |
|  | | E | HK1-vector vs. HK1-INSL5, F1,6BP: P=0.3382;  HK1-vector vs. HK1-INSL5, DHAP: P=0.6125  HK1-vector vs. HK1-INSL5, F-6-P: P=0.0044  HK1-vector vs. HK1-INSL5, G3P: P=0.0219  HK1-vector vs. HK1-INSL5, PEP: P=0.0885  HK1-vector vs. HK1-INSL5, 3-PG: P=0.0333 | |  |
|  | | I | CNE2-vector vs. CNE2-INSL5: P=0.0067;  HK1-vector vs. HK1-INSL5: P=0.0105 | |  |
|  | J | | | CNE2-vector vs. CNE2-INSL5: P=0.0365  HK1-vector vs. HK1-INSL5: P=0.0152 | |
|  | K | | | HK1-vector vs. HK1-INSL5: P=0.0171  HK1-vector Oligomycin vs. HK1-INSL5 Oligomycin: P=0.0136  HK1-vector 2-DGvs. HK1-INSL5 2-DG: P=0.0086  CNE2-vector vs. CNE2-INSL5: P=0.0416  CNE2-vector Oligomycin vs. CNE2-INSL5 Oligomycin: P=0.0879  CNE2-vector 2-DGvs. CNE2-INSL5 2-DG: P=0.0097 | |
|  | L | | | CNE2-vector vs. CNE2-INSL5: P=0.002  HK1-vector vs. HK1-INSL5: P=0.0241 | |
|  | M | | | INSL5(-) vs. INSL5 (+): P=0.0364  INSL5(+) siNC vs INSL5(+) siGPCR142 1#: P=0.0377  INSL5(+) siNC vs INSL5(+) siGPCR142 2#: P=0.0266 | |
|  | N | | | CNE2-EBV siNC vs. CNE2-EBV siINSL5 1#, ATP: P=0.0006; CNE2-EBV siNC vs. CNE2-EBV siINSL5 2#, ATP: P=0.0018  CNE2-EBV siNC vs. CNE2-EBV siINSL5 1#, HK2: P=0.0096; CNE2-EBV siNC vs. CNE2-EBV siINSL5 1#, HK2: P=0.0059  CNE2-EBV siNC vs. CNE2-EBV siINSL5 1#, Lac: P=0.0217; CNE2-EBV siNC vs. CNE2-EBV siINSL5 1#, Lac: P=0.016 | |
| Fig EV4 | F | | | CNE1-vector vs. CNE1-INSL5, 5-FU: P=0.0086; CNE1-vector vs. CNE1-INSL5, DDP: P=0.0092;  CNE2-vector vs. CNE2-INSL5, 5-FU: P=0.0243; CNE2-vector vs. CNE2-INSL5, DDP: P=0.0285;  HK1-vector vs. HK1-INSL5, 5-FU: P=0.3422; HK1-vector vs. HK1-INSL5, DDP: P=0.0031; | |
| Fig EV5 | B | | | PBS vs. INSL5, migration: P=0.0029; INSL5+IgG vs. INSL5+αGPCR142-50, migration: P=0.0011; INSL5+IgG vs. INSL5+αGPCR142-50, migration: P<0.0001;  PBS vs. INSL5, invasion: P=0.0003; INSL5+IgG vs. INSL5+αGPCR142-50, invasion: P=0.0008; INSL5+IgG vs. INSL5+αGPCR142-50, invasion: P=0.0001; | |
|  | E | | | HK1-vector+IgG+DDP vs. HK1-INSL5+IgG+DDP: P=0.0155;  HK1-INSL5+IgG+DDP vs. HK1-INSL5+αINSL5+DDP: P=0.0012;  HK1-INSL5+IgG+DDP vs. HK1-INSL5+αGPCR142+DDP: P=0.0006; | |
|  | F | | | HK1-vector+IgG+DDP vs. HK1-INSL5+IgG+DDP: P<0.0001;  HK1-INSL5+IgG+DDP vs. HK1-INSL5+αINSL5+DDP: P<0.0001;  HK1-INSL5+IgG+DDP vs. HK1-INSL5+αGPCR142+DDP: P<0.0001; | |
