# Supplementary material for: Autocrine INSL5 promotes tumor progression and glycolysis via activation of STAT5 signaling
Source: EMBO Mol Med. 2020 Jul 12;12(9):e12050. doi: 10.15252/emmm.202012050 (PMC7507000; doi:10.15252/emmm.202012050)
Supplement: Supplementary file 2 — Expanded View Figures PDF [file EMMM-12-e12050-s002.pdf]

Expanded View Figures

Figure EV1. EBV-induced INSL5 is highly expressed in NPC and associated with poor prognosis.

A Heat map of upregulated metabolism-associated genes after EBV infection.
B mRNA expression of INSL5 in EBV-positive and EBV-negative NPC cell lines.
C Quantitative RT–PCR showing the expression of INSL5 mRNA in normal nasopharyngeal tissues (NPN) and tumor tissues (NPC).
D Representative immunohistochemical (IHC) staining of INSL5 in NPC paraffin samples. Scale bars represent 100 μm.
E–I Kaplan–Meier curves showing the impact of INSL5 expression on overall survival for glioma, kidney renal clear cell carcinoma, sarcoma, uterine carcinosarcoma, and uveal melanoma (GEPIA database). Analysis of TCGA data indicates that high expression of INSL5 was correlated with poor prognosis in these cancer types.
J–K The calibration curve of nomogram for predicting overall survival (OS) at 5 years (J) and predicting disease-free survival (DFS) at 5 years (K). Actual OS or DFS is plotted on the *y*-axis; nomogram predicted probability of OS or DFS is plotted on the *x*-axis.

Data information: In (B), data are presented as mean ± SEM, in (C), data are presented as mean ± SD, from three different experiments, and *P*-values were determined by unpaired *t*-test. *$P < 0.05$, **$P < 0.01$, ***$P < 0.001$, ns, no significance. Exact *P*-values are specified in Appendix Table S4.

Source data are available online for this figure.

 © 2020 The Authors

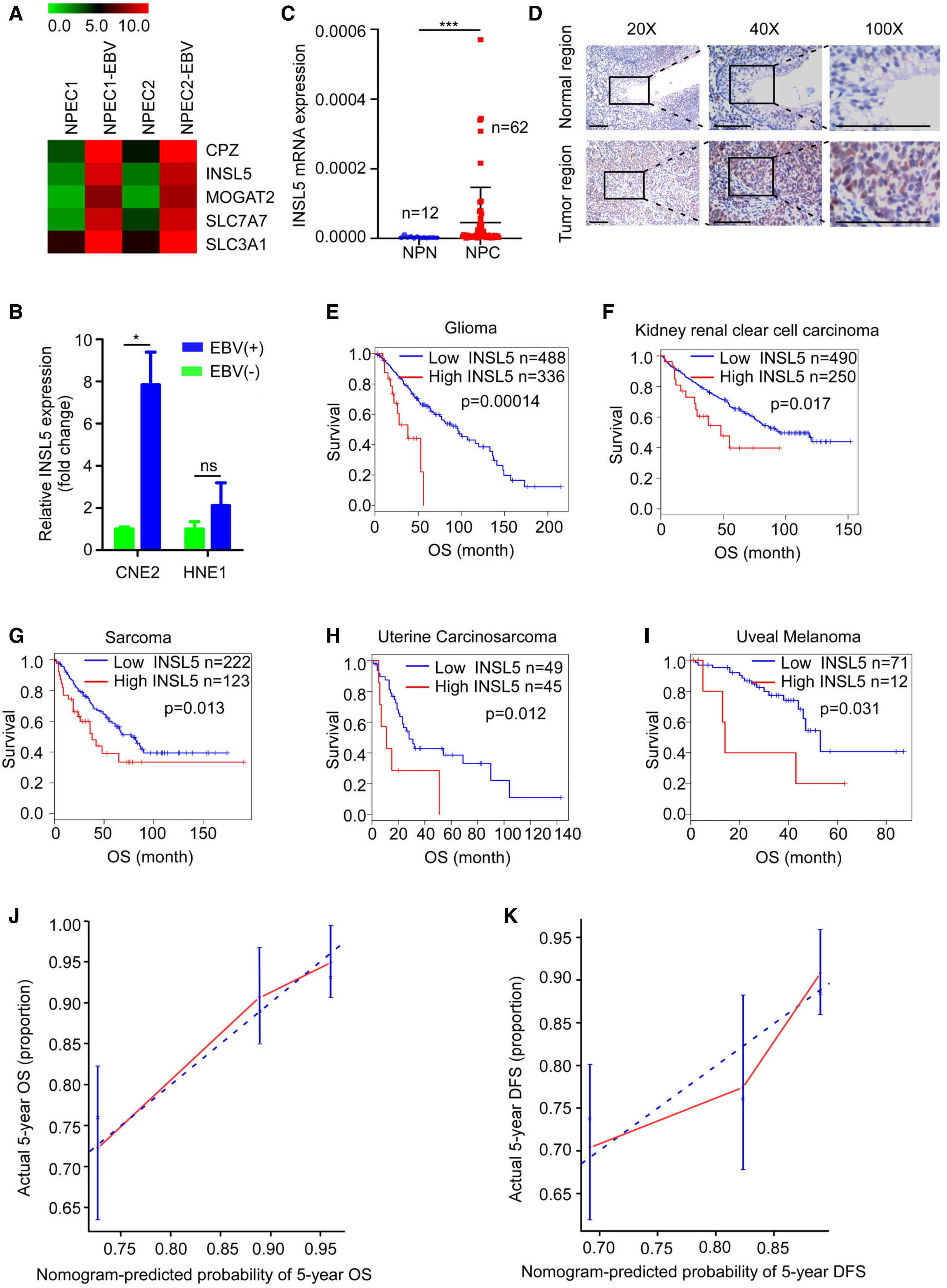

A
0.0 5.0 10.0
NPEC1
NPEC1-EBV
NPEC2
NPEC2-EBV
CPZ
INSL5
MOGAT2
SLC7A7
SLC3A1
C
INSL5 mRNA expression
0.0006
0.0004
0.0002
0.0000
***
n=62
n=12
NPN
NPC
D
20X
40X
100X
Normal region
Tumor region
B
Relative INSL5 expression (fold change)
10
8
6
4
2
0
*
ns
EBV(+)
EBV(-)
CNE2
HNE1
E
Glioma
Low INSL5 n=488
High INSL5 n=336
p=0.00014
Survival
OS (month)
F
Kidney renal clear cell carcinoma
Low INSL5 n=490
High INSL5 n=250
p=0.017
G
Sarcoma
Low INSL5 n=222
High INSL5 n=123
p=0.013
H
Uterine Carcinosarcoma
Low INSL5 n=49
High INSL5 n=45
p=0.012
I
Uveal Melanoma
Low INSL5 n=71
High INSL5 n=12
p=0.031
J
Actual 5-year OS (proportion)
Nomogram-predicted probability of 5-year OS
K
Actual 5-year DFS (proportion)
Nomogram-predicted probability of 5-year DFS

Figure EV1.

Figure EV2. INSL5 overexpression promotes the progression of NPC via accelerating cell proliferation and invasion depending on GPCR142.

A Exogenous expression of INSL5 in normal immortalized NPEC cell lines.

B Quantitative RT–PCR validation for siRNA knockdown efficiency of GPCR142 in INSL5 stably overexpressing CNE1, CNE2, and HK1cell lines.

C–F MTT (C), colony formation (D), Brdu incorporation (E), migration, and invasion assay (F) of vector control or INSL5 overexpressing NP69 cell line. The scale bars represent 20 μm in (E) and 100 μm in (F).

G Invasion assays of vector control or INSL5 overexpressing CNE1, CNE2, and HK1 NPC cell lines either transfected with control siRNA (NC) or GPCR142 siRNA (#1 and #2). Representative images are shown in left. Invaded cells per field of view were plotted in right. The scale bars represent 100 μm.

H EBV-positive NPC cell lines CNE2-EBV and HNE1-EBV were transfected with siRNA against INSL5 for 36 h, followed by immunoblotting with indicated antibodies (inserted picture). MTT assay of CNE2-EBV (upper panel) and HNE1-EBV (lower panel) cells after INSL5 knockdown.

I Migration and invasion assay of CNE2-EBV (upper) and HNE1-EBV (lower) cells with INSL5 knockdown. Representative images are shown in left panels. Migrated or invaded cells per field of view were plotted in right panels. The scale bars represent 100 μm.

J Colony formation assay of CNE2-EBV and HNE1-EBV cells with INSL5 knockdown. Representative images are shown in left panels. Number of colonies was plotted in right panels.

K, L Xenograft tumor growth of INSL5 overexpression NPC CNE2 stable cell lines in nude mice. Tumor size (K) and tumor weight of two groups (L), $n = 7$ mice per group.

Data information: In (B and G–L), data are presented as mean ± SEM, in (C–F), data are presented as mean ± SD, from three different experiments, and P-values were determined by unpaired t-test. *$P < 0.05$, **$P < 0.01$, ***$P < 0.001$, ns, no significance. Exact P-values are specified in Appendix Table S4.

Source data are available online for this figure.

 © 2020 The Authors

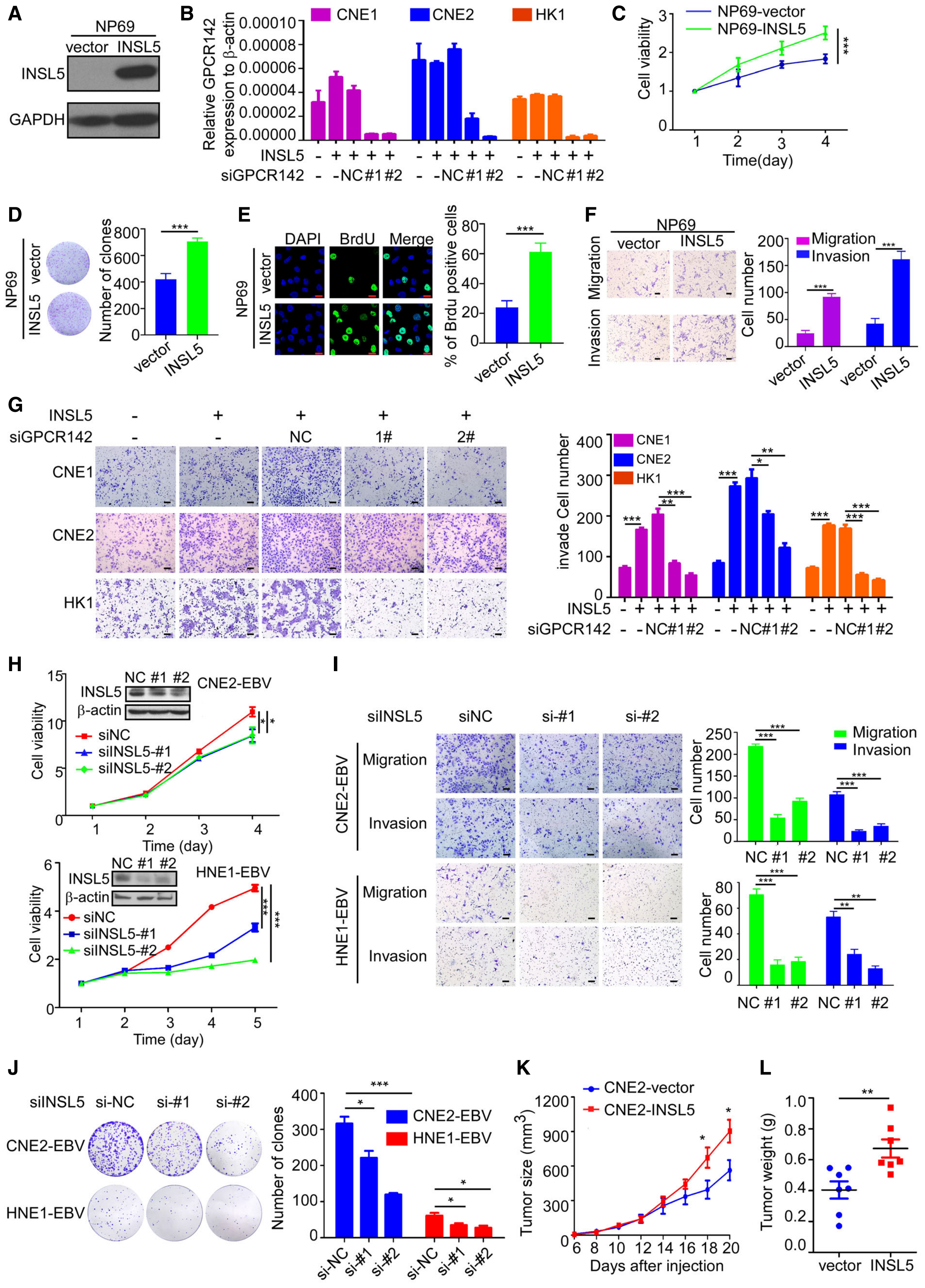
A
NP69
vector INSL5
INSL5
GAPDH
B
CNE1 CNE2 HK1
Relative GPCR142 expression to β-actin
INSL5
siGPCR142
C
NP69-vector
NP69-INSL5
Cell viability
Time(day)
D
NP69
vector
INSL5
Number of clones
E
DAPI BrdU Merge
% of Brdu positive cells
F
Migration
Invasion
Cell number
G
invade Cell number
H
CNE2-EBV
HNE1-EBV
siNC
siINSL5-#1
siINSL5-#2
β-actin
Time (day)
I
siINSL5 siNC si-#1 si-#2
J
Number of clones
K
CNE2-vector
CNE2-INSL5
Tumor size (mm3)
Days after injection
L
Tumor weight (g)

Figure EV2.

Figure EV3. INSL5 induces glucose metabolism to aerobic glycolysis reprogramming in NPC cells.

A Analysis of glycolytic gene expression by qRT–PCR in INSL5 overexpressed CNE1 cell lines.
B Analysis of glycolytic gene expression by immunoblot in INSL5 overexpressing cells with or without GPCR142 knockdown.
C–E The metabolomics identified increased glycolytic intermediate metabolites in INSL5 overexpressed CNE1 cells (C), CNE2 cells (D), and HK1 cells (E). CNE1 and HK1 were from five independent samples, and CNE2 were from seven independent samples.
F Schematic diagram of aerobic glycolysis pathway and TCA pathway. Red: INSL5 upregulated glycolytic genes and metabolites. Green: INSL5 downregulated TCA intermediates.
G The extracellular acidification rate (ECAR) was measured in cells with or without INSL5 overexpression using a Seahorse XF96 Extracellular Flux analyzer.
H The oxygen consumption rate (OCR) was measured in cells with or without INSL5 overexpression using a Seahorse XF96 Extracellular Flux analyzer.
I–L Glucose uptake (I), HK2 enzyme activity (J), ATP concentration (K), and lactate production (L) in CNE2 and HK1 stable cells.
M Glucose uptake in CNE2 wide-type or GPCR142 knockdown cells stimulated with INSL5 peptide (50 ng/ml) for 24 h.
N ATP concentration, HK2 enzyme activity, and lactate production in INSL5 wide-type or knockdown CNE2-EBV cells.

Data information: In (G–I, M and N), data are presented as mean ± SEM, in (C–E and J–L), data are presented as mean ± SD, from three different experiments, and *P*-values were determined by unpaired t-test. **P* < 0.05, ***P* < 0.01, ****P* < 0.001, ns, no significance. Exact *P*-values are specified in Appendix Table S4.

Source data are available online for this figure.

© 2020 The Authors

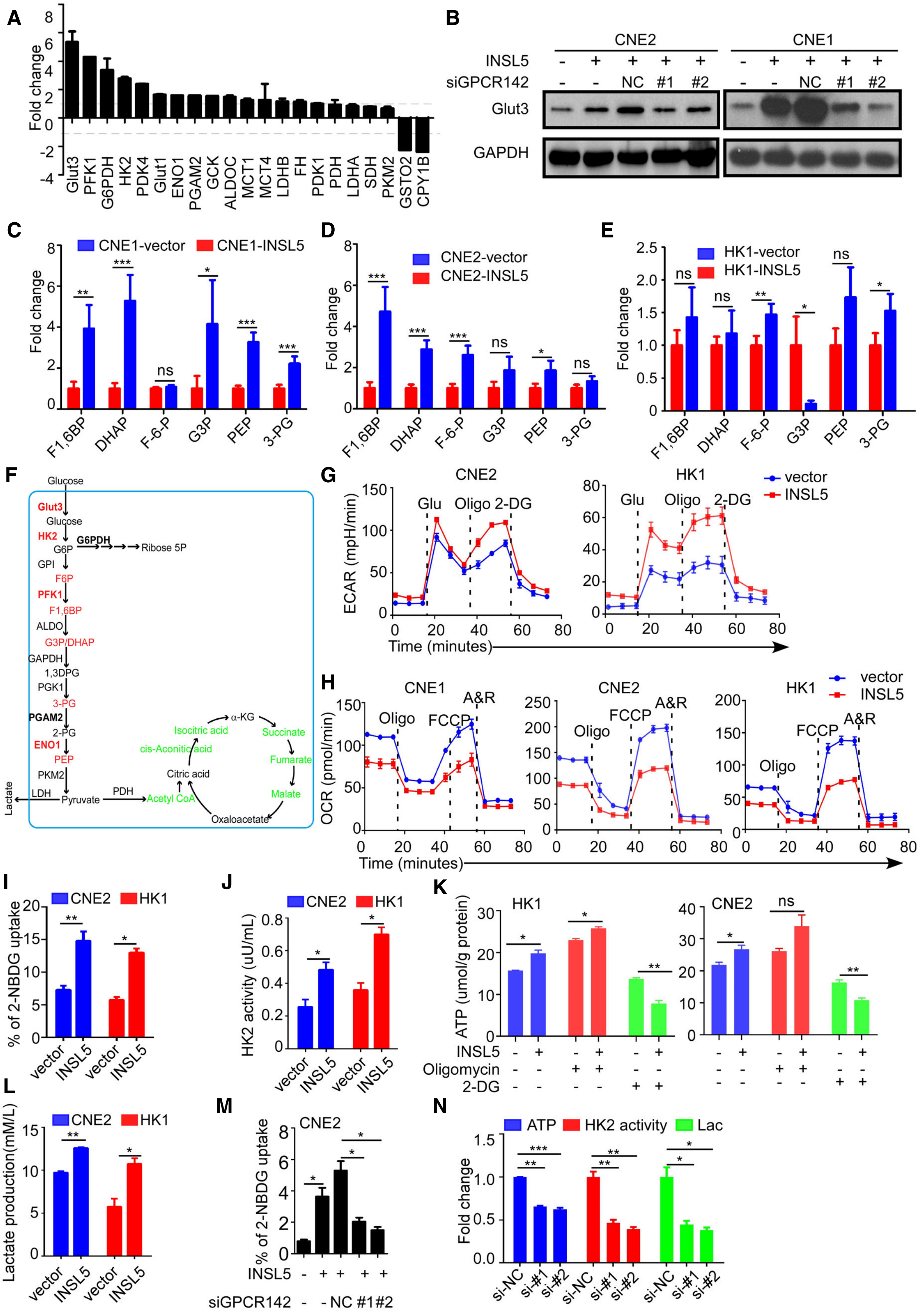

A
B
C
D
E
F
G
H
I
J
K
L
M
N
CNE2
CNE1
INSL5
siGPCR142
Glut3
GAPDH
CNE1-vector
CNE1-INSL5
CNE2-vector
CNE2-INSL5
HK1-vector
HK1-INSL5
Fold change
Glucose
Ribose 5P
Pyruvate
Lactate
Acetyl CoA
Citric acid
Oxaloacetate
ECAR (mpH/min)
OCR (pmol/min)
Time (minutes)
vector
Glu
Oligo
2-DG
FCCP
A&R
% of 2-NBDG uptake
HK2 activity (uU/mL)
ATP (umol/g protein)
Oligomycin
Lactate production(mM/L)
ATP
HK2 activity
Lac

Figure EV3.

Figure EV4. Overexpression of INSL5 promotes cell cycle progression and suppresses cell apoptosis.

A The cell cycle of vector control or INSL5 overexpressing CNE1, CNE2, and HK1 cells were analyzed by flow cytometry assay.
B Statistical analysis of cell percentage in each cell cycle phase.
C Cyclin D, cyclin E, cyclin B, and p27 expression levels were detected by Western blotting in cells with or without INSL5 overexpression.
D Western blotting for c-myc, BCL2, and BCL-xL in CNE1, CNE2, and HK1 with or without INSL5 overexpression.
E CNE1 stable cell line was treated with DDP or 5-FU and stained with annexin V/propidium iodide (PI), and measured by flow cytometry. Data shown are representative of three independent experiments.
F Statistical analysis of the effects of INSL5 overexpression on cell apoptosis under DDP or 5-FU treatment.
G Western blotting for apoptosis pathway in CNE1, CNE2, and HK1 with or without INSL5 overexpression under 5-FU treatment.

Data information: In (F), data are presented as mean $\pm$ SEM, from three different experiments, and P-values were determined by unpaired t-test. *$P < 0.05$, **$P < 0.01$, ***$P < 0.001$, ns, no significance. Exact P-values are specified in Appendix Table S4.

Source data are available online for this figure.

© 2020 The Authors

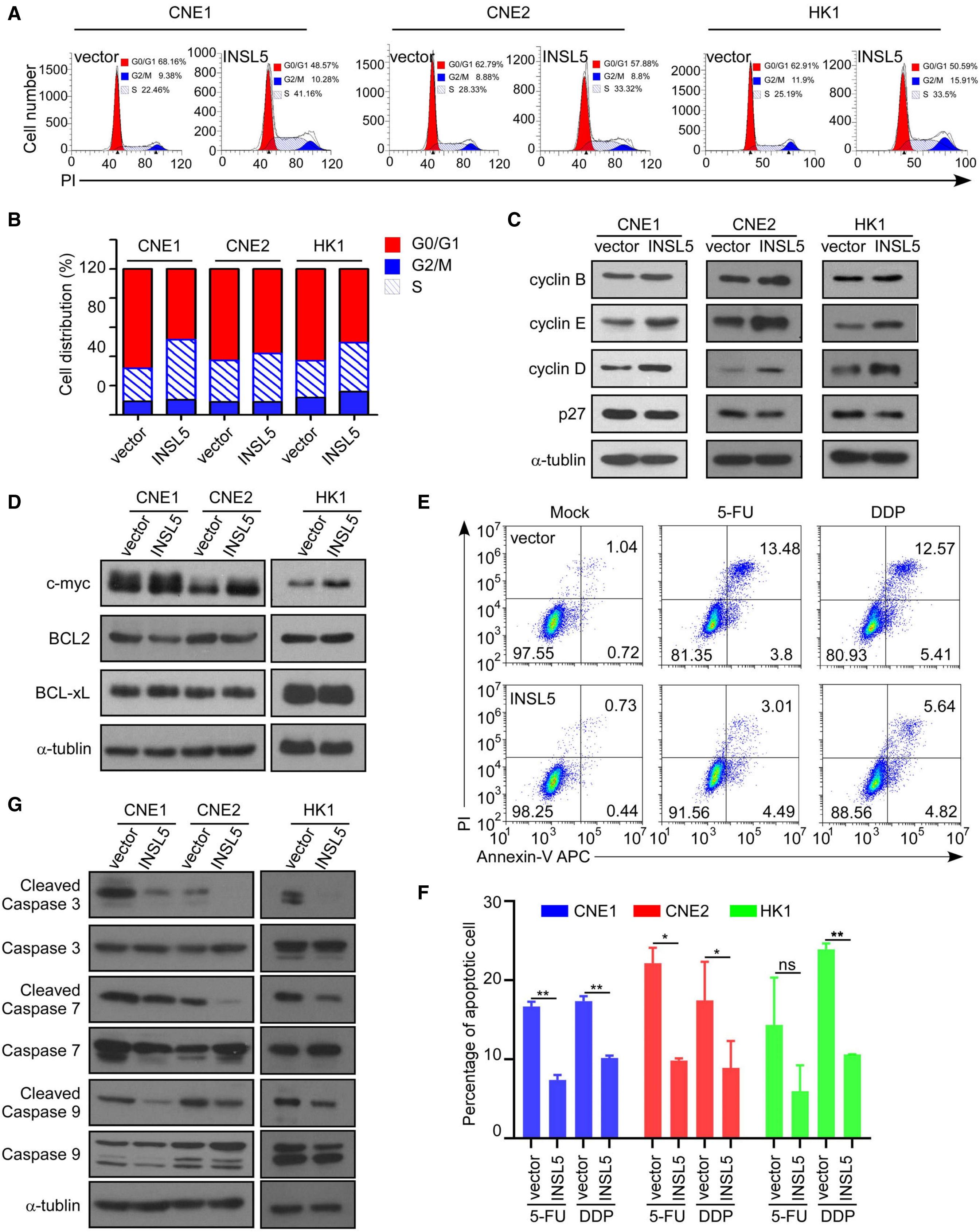
A
CNE1
CNE2
HK1
Cell number
PI
vector
INSL5
B
Cell distribution (%)
G0/G1
G2/M
S
C
cyclin B
cyclin E
cyclin D
p27
α-tublin
D
c-myc
BCL2
BCL-xL
E
Mock
5-FU
DDP
Annexin-V APC
F
Percentage of apoptotic cell
ns
G
Cleaved Caspase 3
Caspase 3
Cleaved Caspase 7
Caspase 7
Cleaved Caspase 9
Caspase 9

Figure EV4.

Figure EV5. Overexpression of INSL5 sensitizes NPC to GPCR142 neutralized antibody.

A CNE1 cells stimulated with 100 ng/ml INSL5 peptide in the presence of an anti-INSL5 antibody at 50 or 100 μg/ml, or an isotype-matched IgG control (IgG) for 24 h. After that, cell was stained with annexin V/propidium iodide (PI), and measured cell death and apoptosis by flow cytometry.

B Migration and invasion assay for CNE1 cells stimulated with 50 ng/ml INSL5 peptide in the presence or absence of an anti-GPCR142 antibody at 50 or 100 μg/ml, or an isotype-matched IgG control (IgG) for 24 h100 μm (left panel), and the statistical analysis of cell migration and invasion (right panel).

C Growth curves of cell lines stably expressing INSL5 or vector 48 h post-treatment with the indicated dose of 5-FU and 2-DG.

D The tumor image of anti-INSL5, anti-GPCR142, or isotype-matched IgG control (IgG)-treated PDX.

E–G Tumor weight (E), tumor size (F), and tumor image (G) of nude mice burdened with HK1vector/INSL5 tumor and intraperitoneally injected with anti-INSL5, anti-GPCR142, or isotype-matched IgG control (IgG) and DDP every 3 days. n = 6 mice per group.

Data information: In (B, C, E and F), data are presented as mean ± SD, in (C–E and J–L), data are presented as mean ± SD, from three different experiments, and P-values were determined by unpaired t-test. *$P < 0.05$, **$P < 0.01$, ***$P < 0.001$, ns, no significance. Exact P-values are specified in Appendix Table S4.

Source data are available online for this figure.

© 2020 The Authors

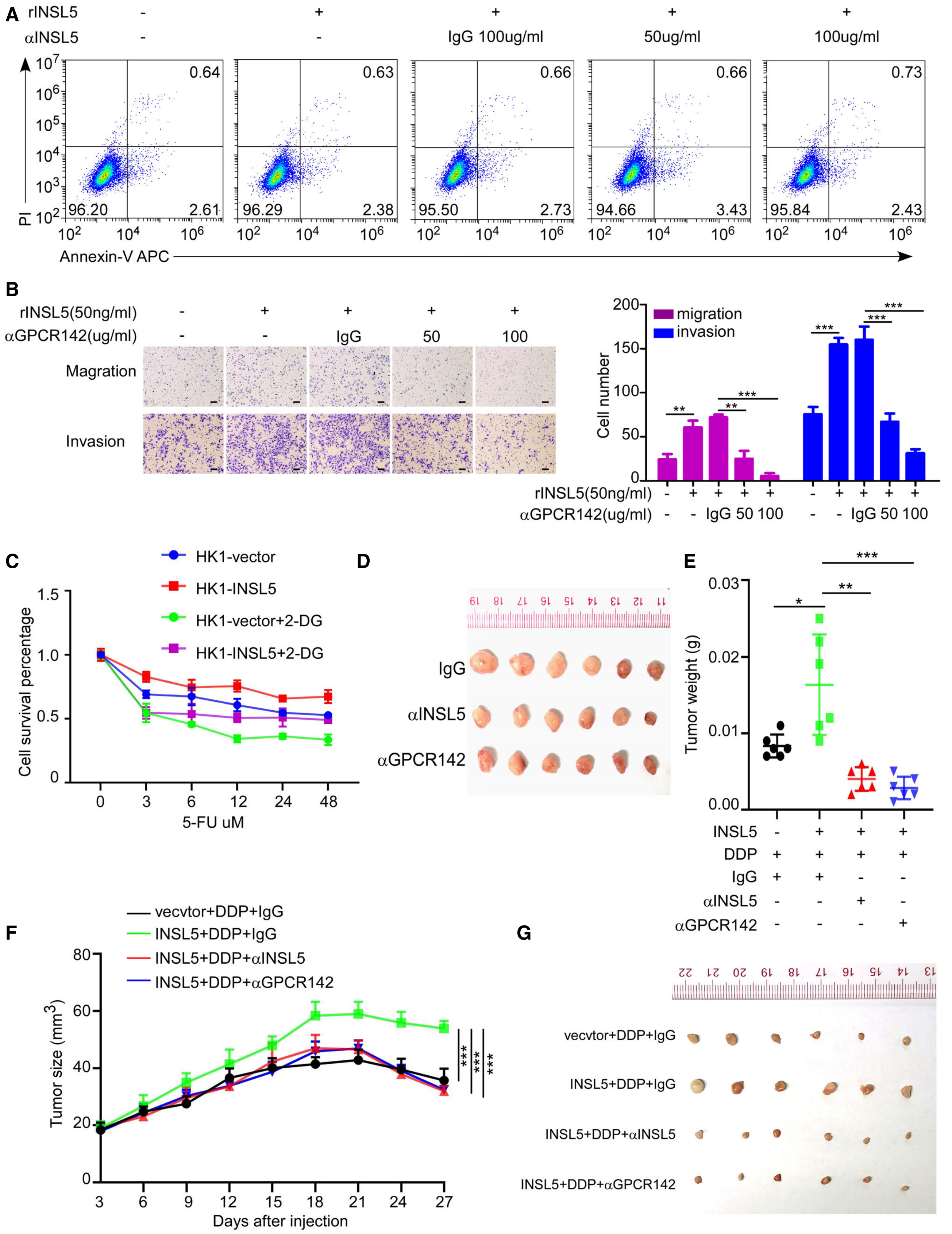

A
rINSL5 - + + + +
αINSL5 - - IgG 100ug/ml 50ug/ml 100ug/ml
0.64 96.20 2.61
0.63 96.29 2.38
0.66 95.50 2.73
0.66 94.66 3.43
0.73 95.84 2.43
PI
Annexin-V APC
B
rINSL5(50ng/ml) - + + + +
αGPCR142(ug/ml) - - IgG 50 100
Magration
Invasion
migration
invasion
Cell number
C
HK1-vector
HK1-INSL5
HK1-vector+2-DG
HK1-INSL5+2-DG
Cell survival percentage
5-FU uM
D
IgG
αINSL5
αGPCR142
E
Tumor weight (g)
INSL5 - + + +
DDP + + + +
IgG + + - -
αINSL5 - - + -
αGPCR142 - - - +
F
vecvtor+DDP+IgG
INSL5+DDP+IgG
INSL5+DDP+αINSL5
INSL5+DDP+αGPCR142
Tumor size (mm3)
Days after injection
G
vecvtor+DDP+IgG
INSL5+DDP+IgG
INSL5+DDP+αINSL5
INSL5+DDP+αGPCR142

Figure EV5.
