# Supplementary figures and images for: Autocrine INSL5 promotes tumor progression and glycolysis via activation of STAT5 signaling
Source: EMBO Mol Med. 2020 Jul 12;12(9):e12050. doi: 10.15252/emmm.202012050 (PMC7507000; doi:10.15252/emmm.202012050)

Fig EV4C

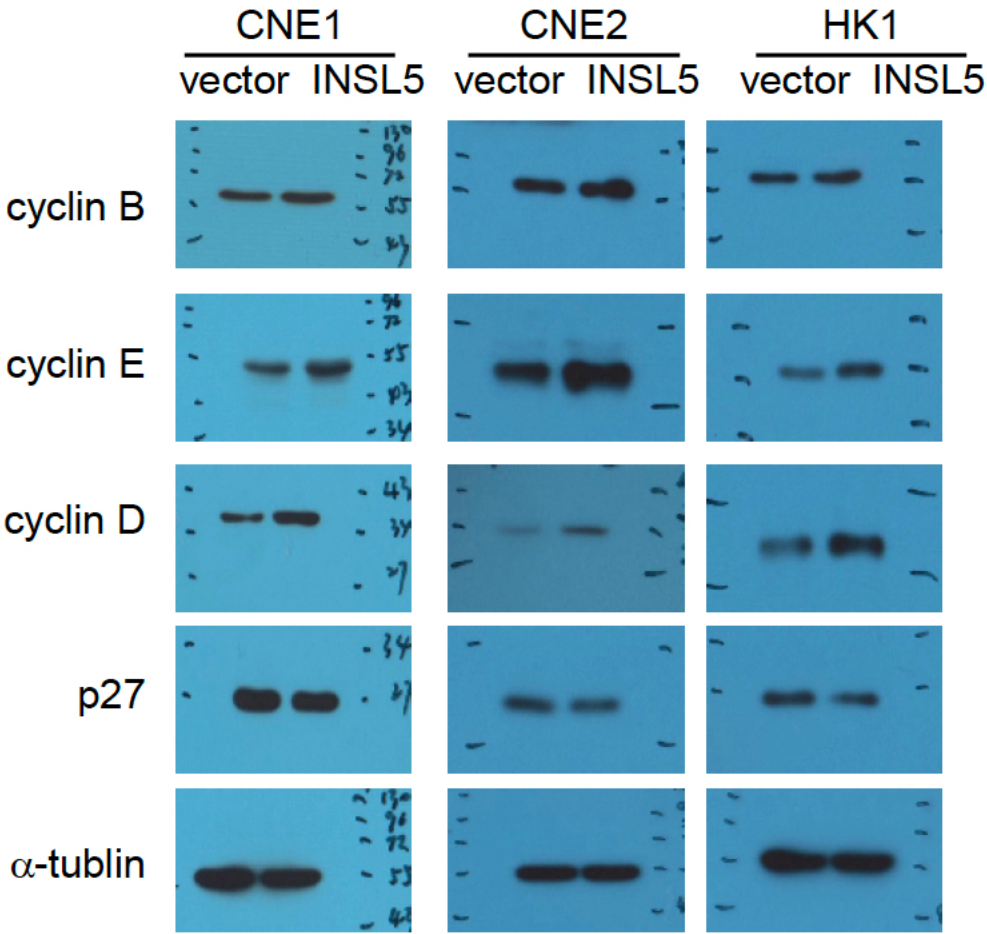

Fig EV4D

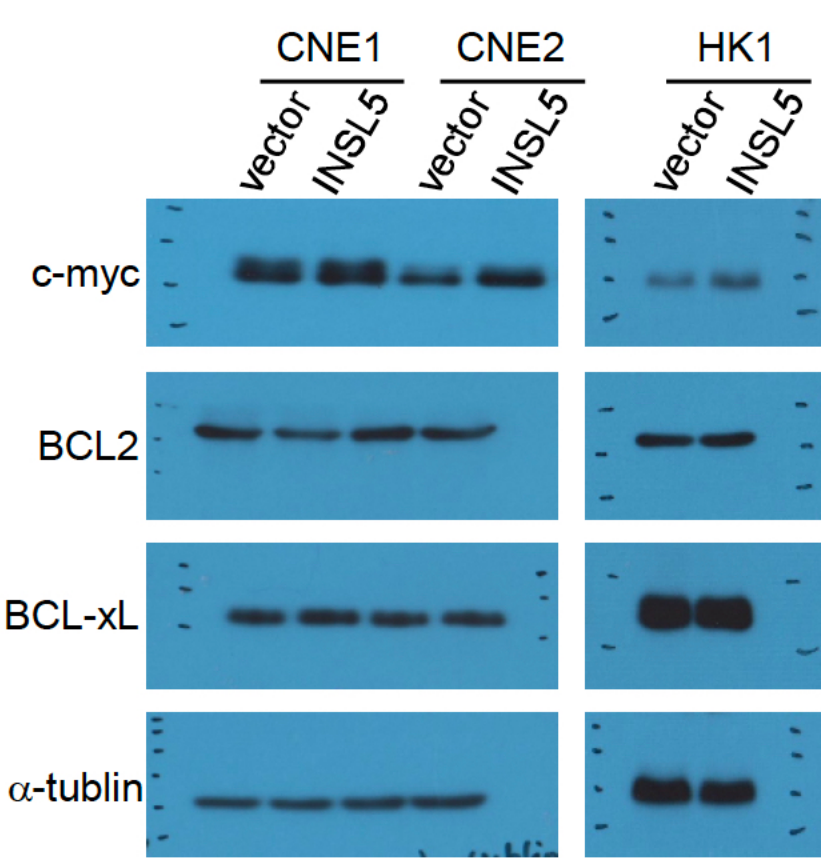

Fig EV4G

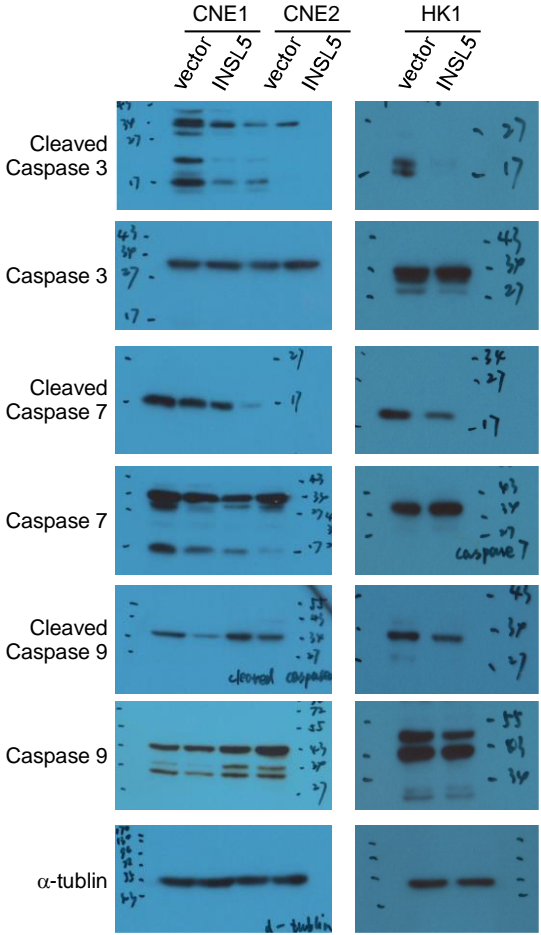

Supplement: Supplementary file 3 — Source Data for Expanded View [file EMMM-12-e12050-s008.zip › Figure EV4 western_blot_Source_Data.pdf]

Fig EV3B

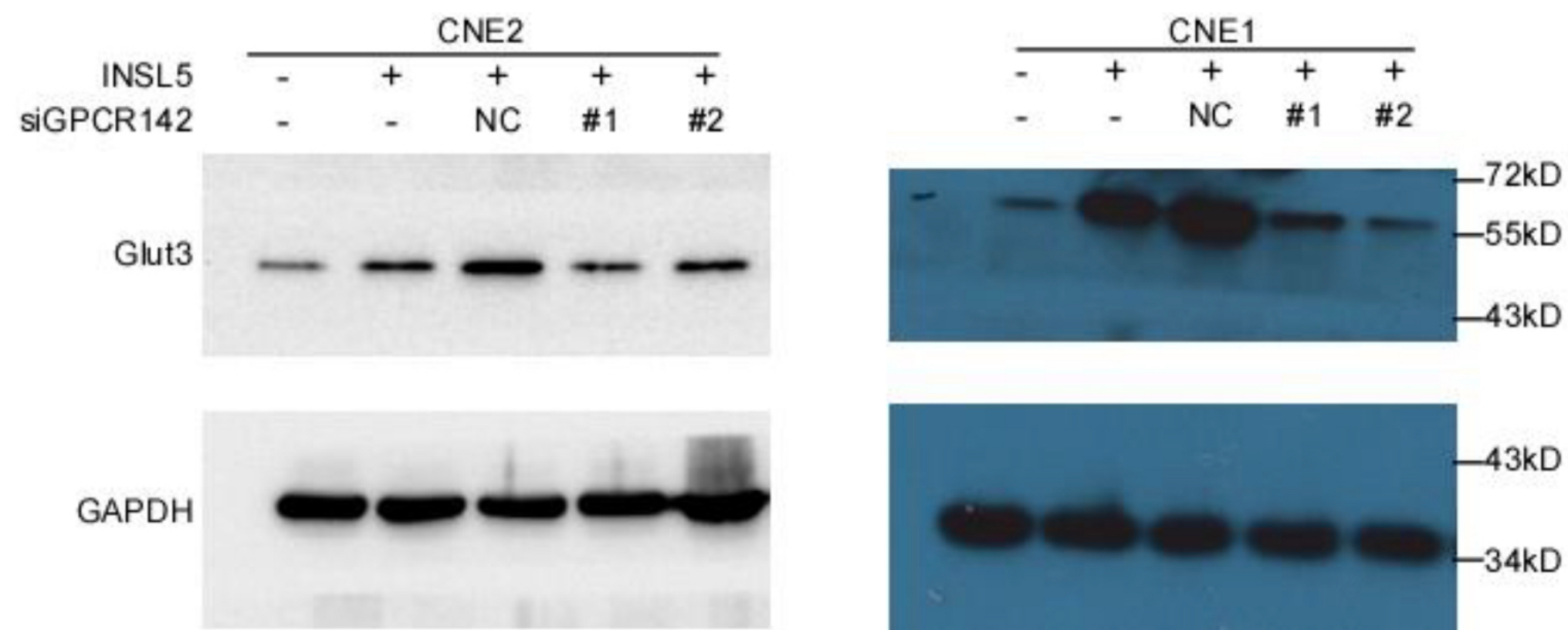

Supplement: Supplementary file 3 — Source Data for Expanded View [file EMMM-12-e12050-s008.zip › Figure EV3 western_blot_Source_Data.pdf]

Figure EV5B

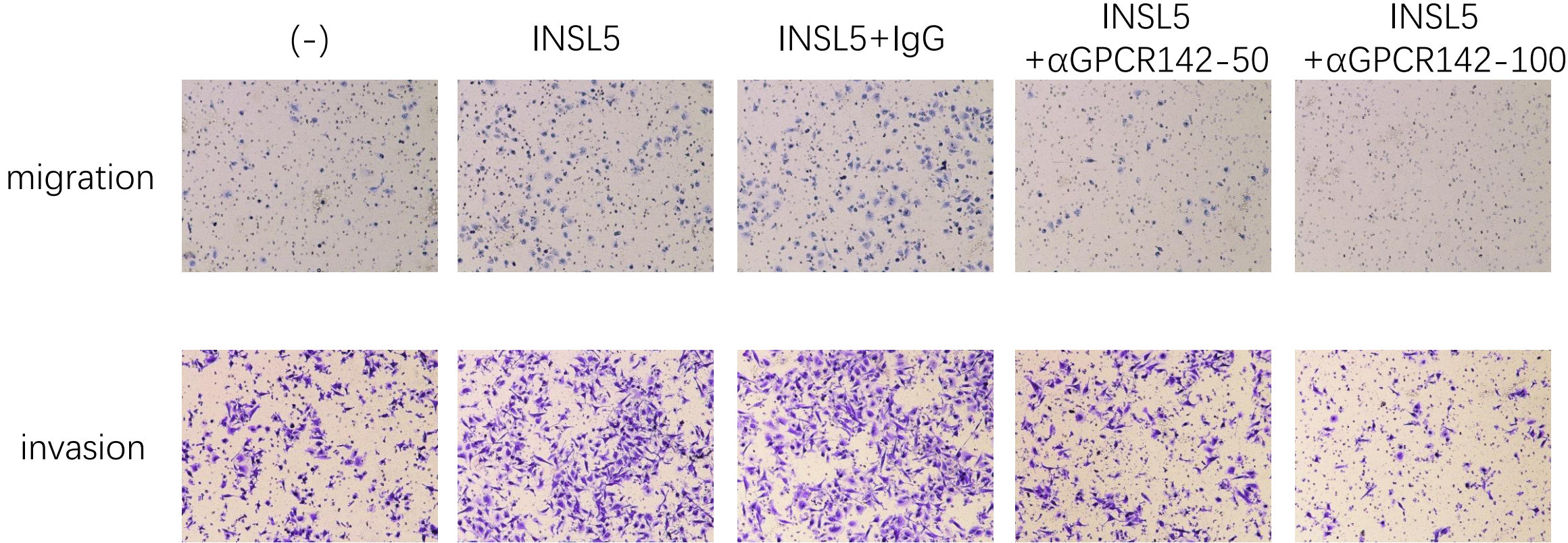

Supplement: Supplementary file 3 — Source Data for Expanded View [file EMMM-12-e12050-s008.zip › Figure EV5 Microscope SD.pdf]

Figure EV1D

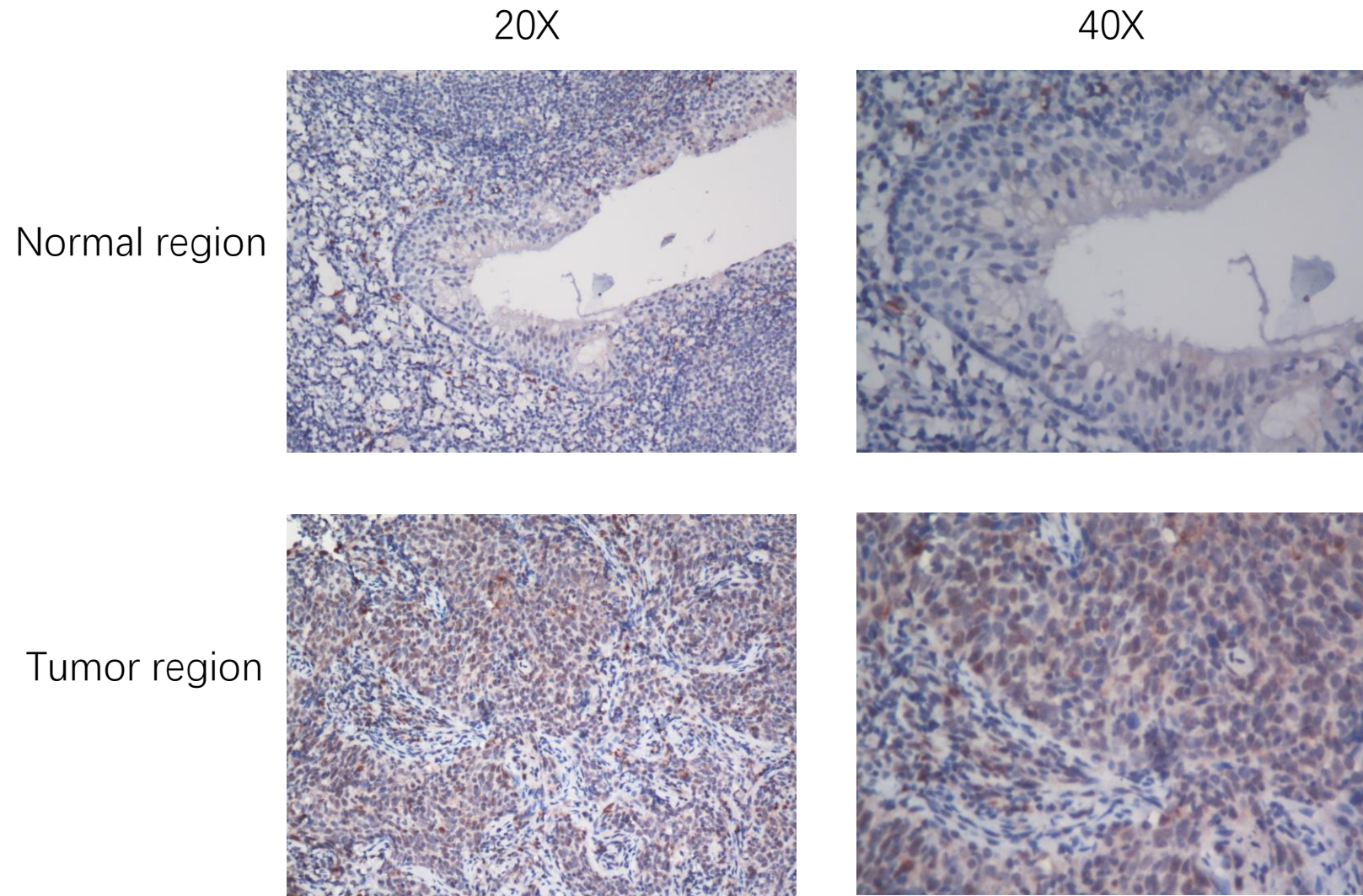

Supplement: Supplementary file 3 — Source Data for Expanded View [file EMMM-12-e12050-s008.zip › Figure EV1 Microscope SD.pdf]

Fig 1A

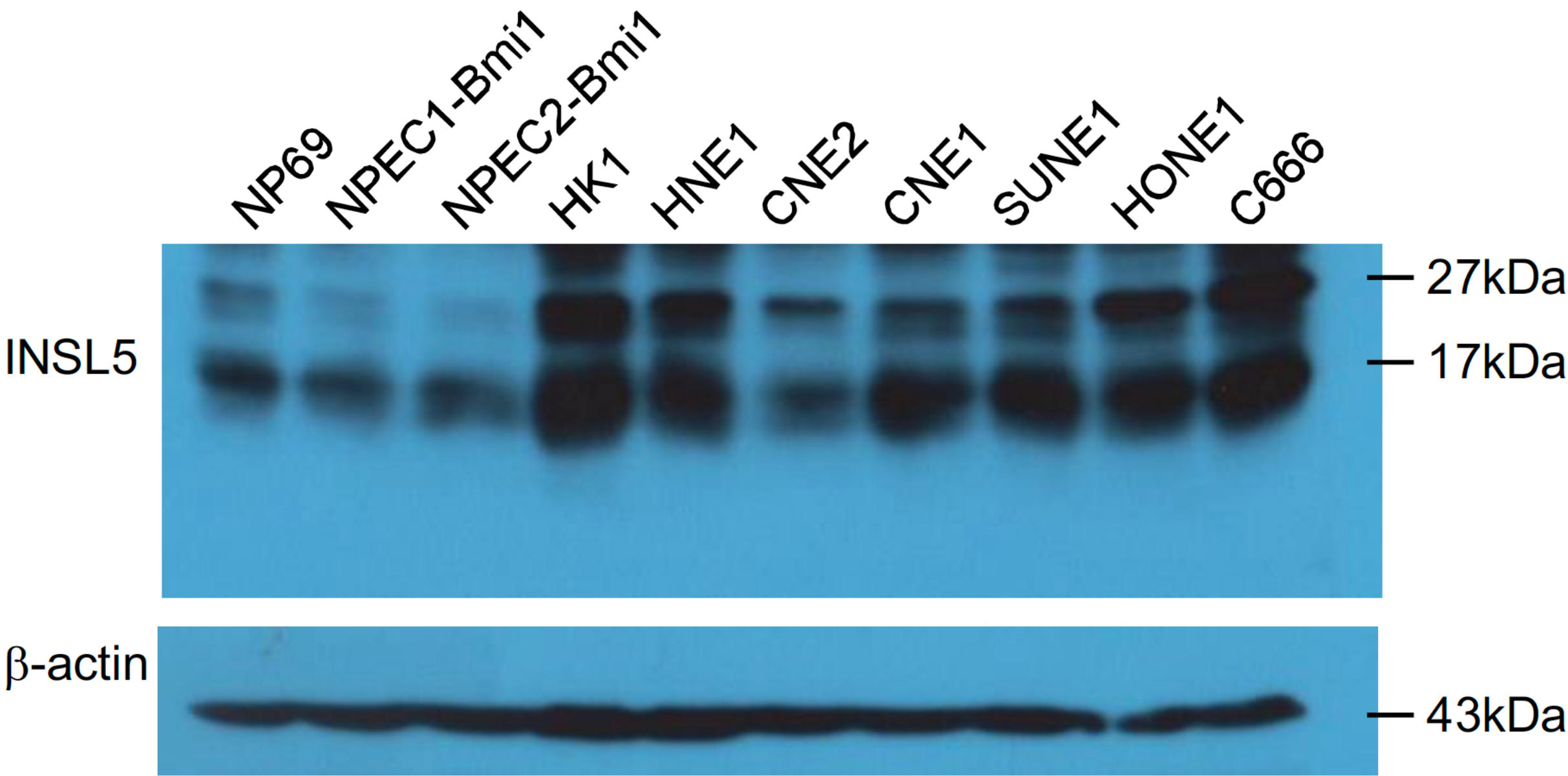

Supplement: Supplementary file 5 — Source Data for Figure 1 [file EMMM-12-e12050-s003.pdf]

Fig 3A

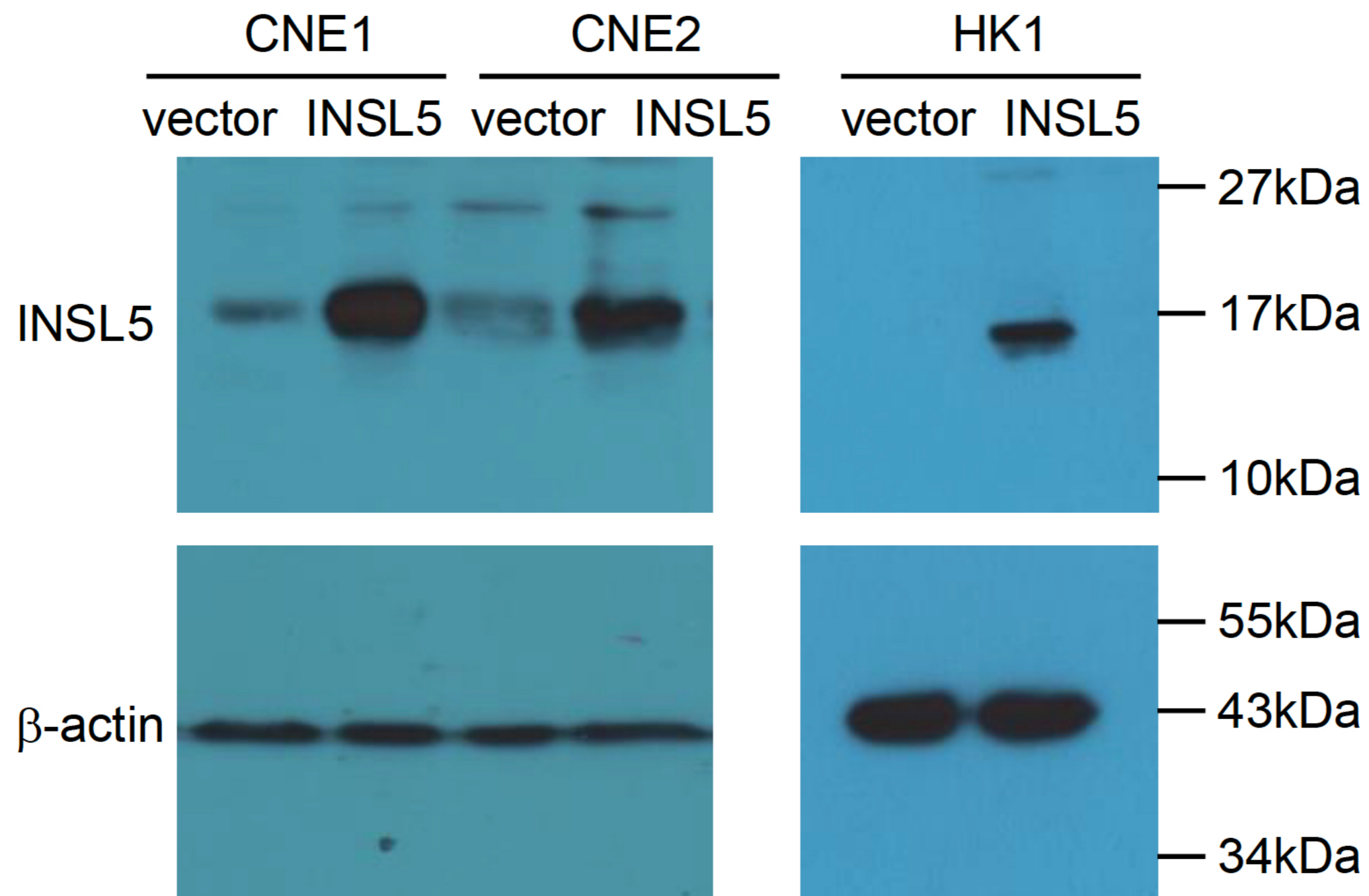

Supplement: Supplementary file 6 — Source Data for Figure 3 [file EMMM-12-e12050-s004.zip › Figure 3 western_blot_Source_Data.pdf]

Figure 3A

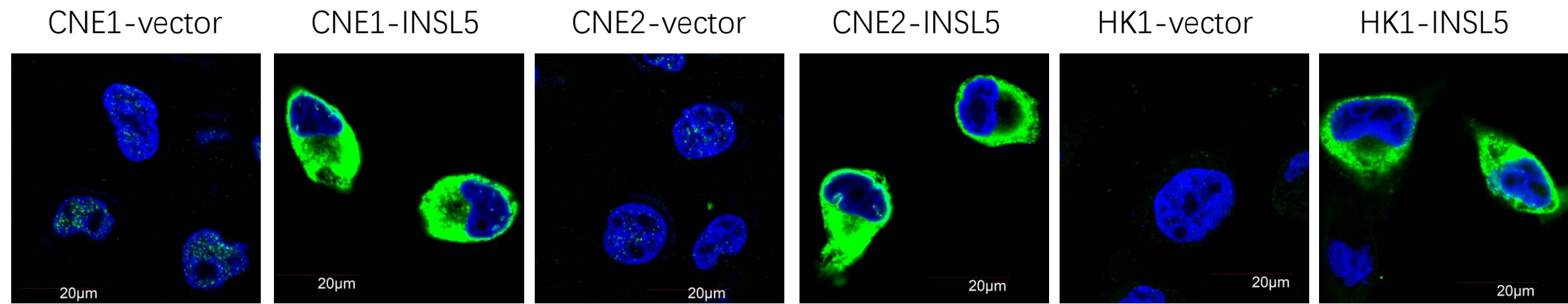

Figure 3E

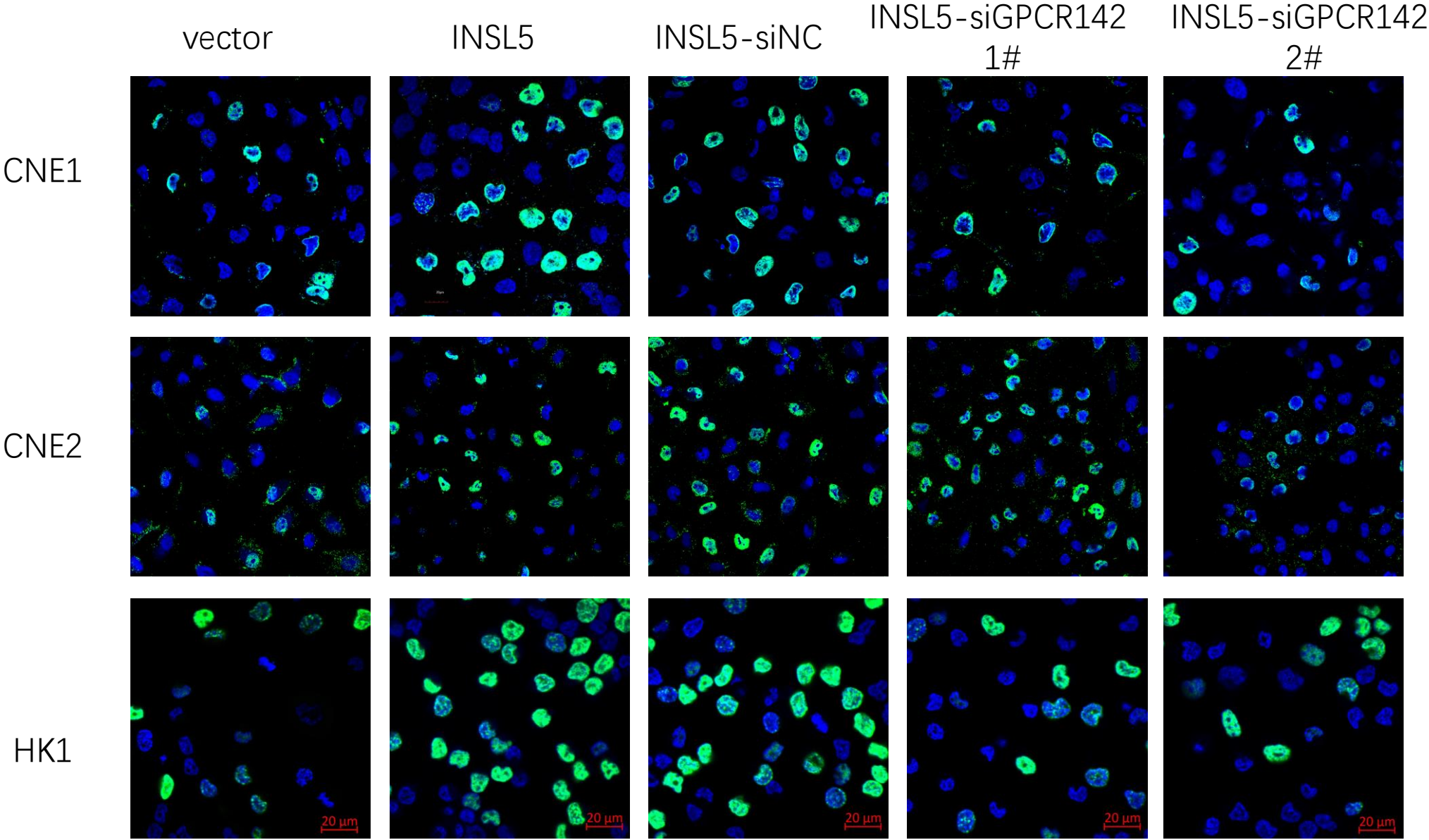

Figure 3G

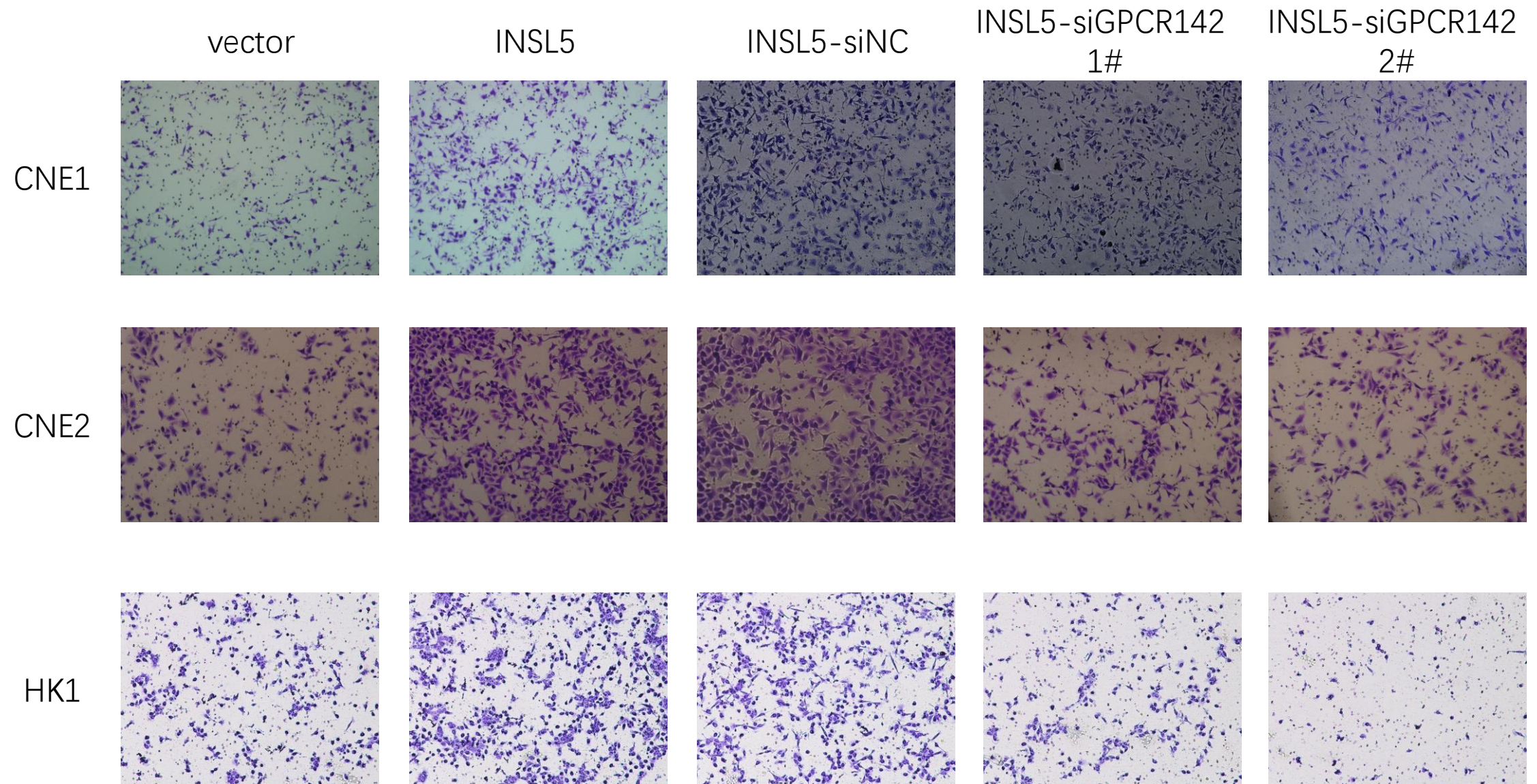

Supplement: Supplementary file 6 — Source Data for Figure 3 [file EMMM-12-e12050-s004.zip › Figure 3 Microscope SD.pdf]

Fig 4D

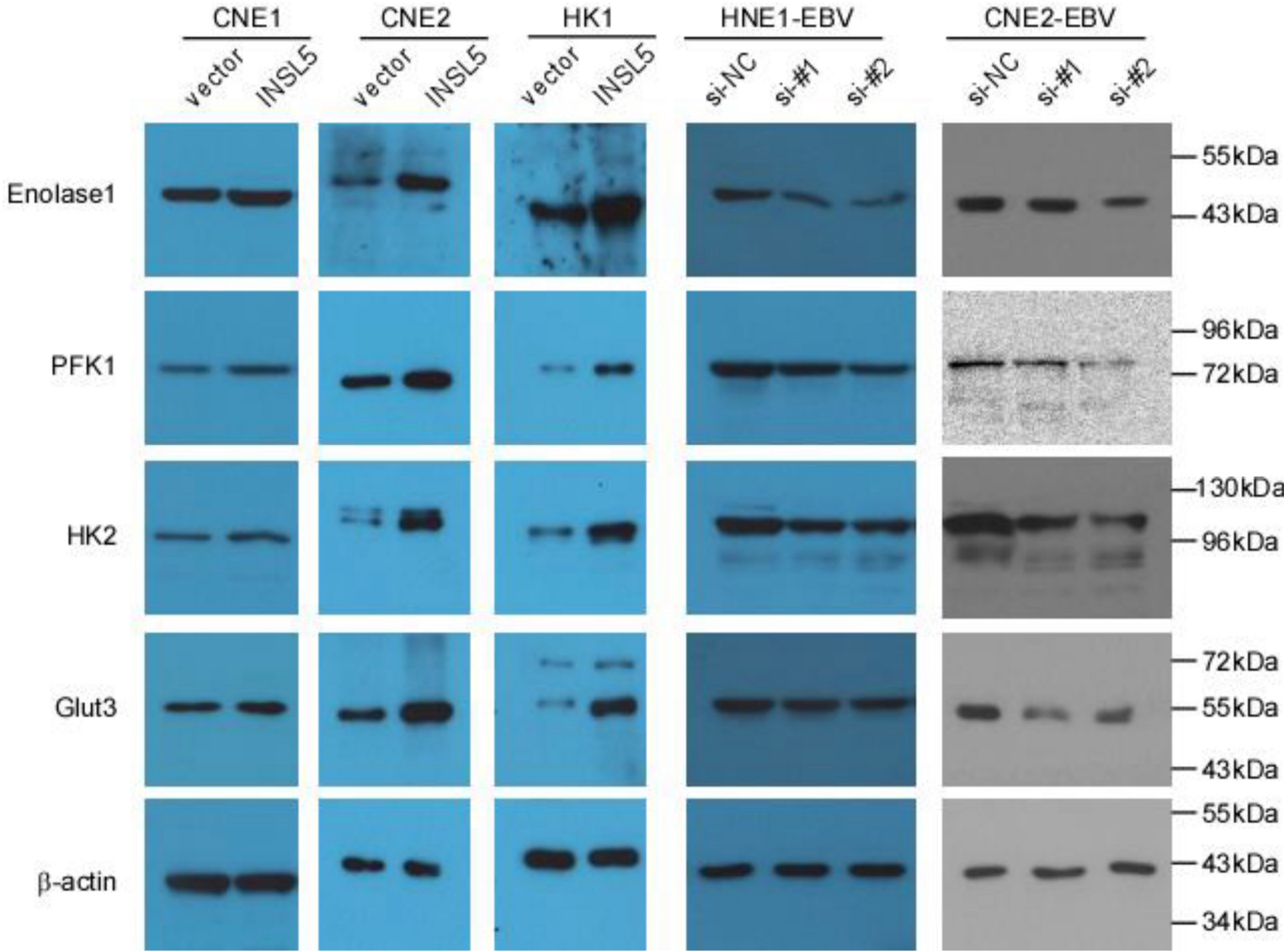

Supplement: Supplementary file 7 — Source Data for Figure 4 [file EMMM-12-e12050-s005.pdf]

Fig 5B and 5D

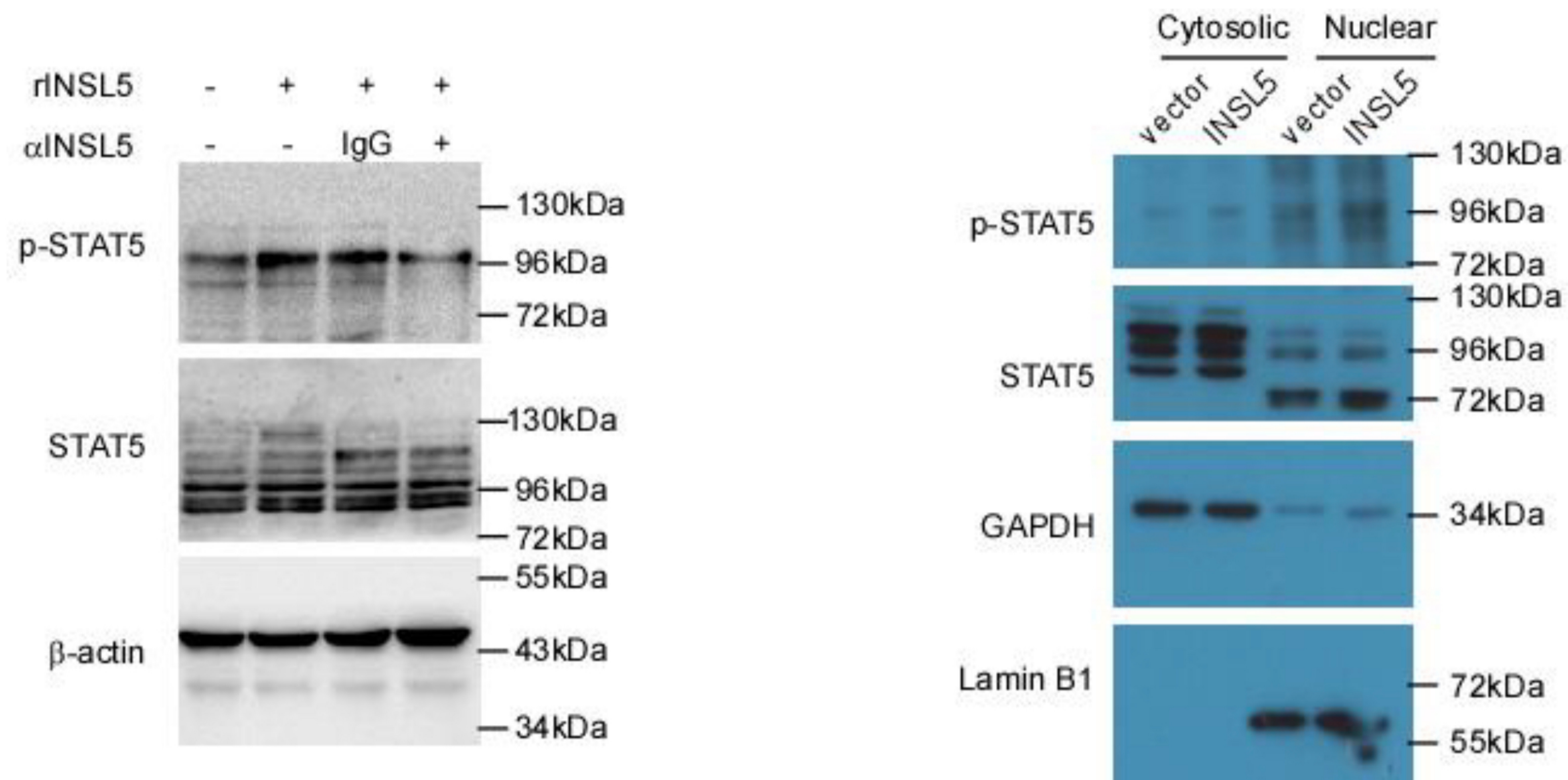

Fig 5E

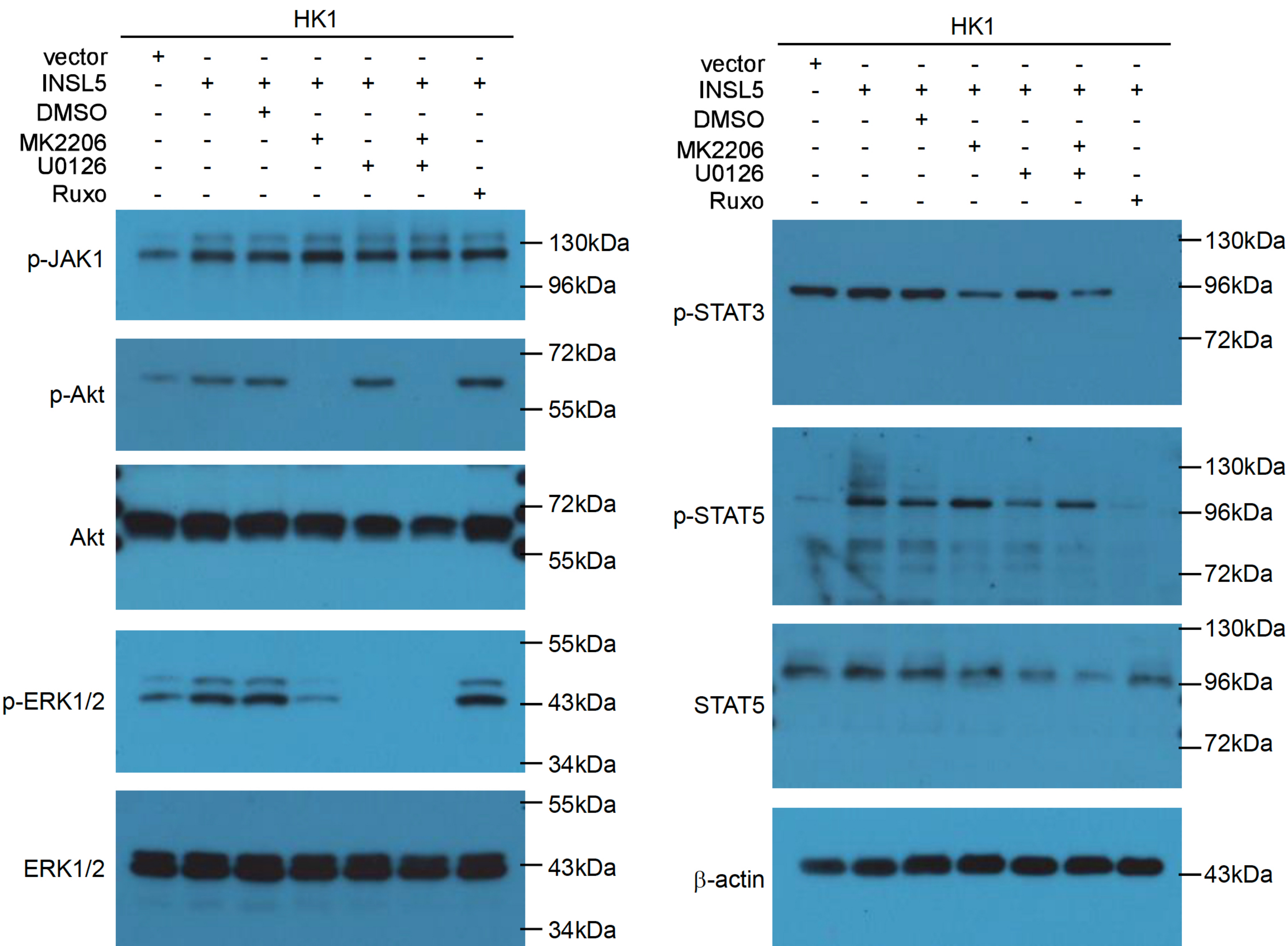

Supplement: Supplementary file 8 — Source Data for Figure 5 [file EMMM-12-e12050-s006.zip › Figure 5 western_blot_Source_Data.pdf]

Figure 5C

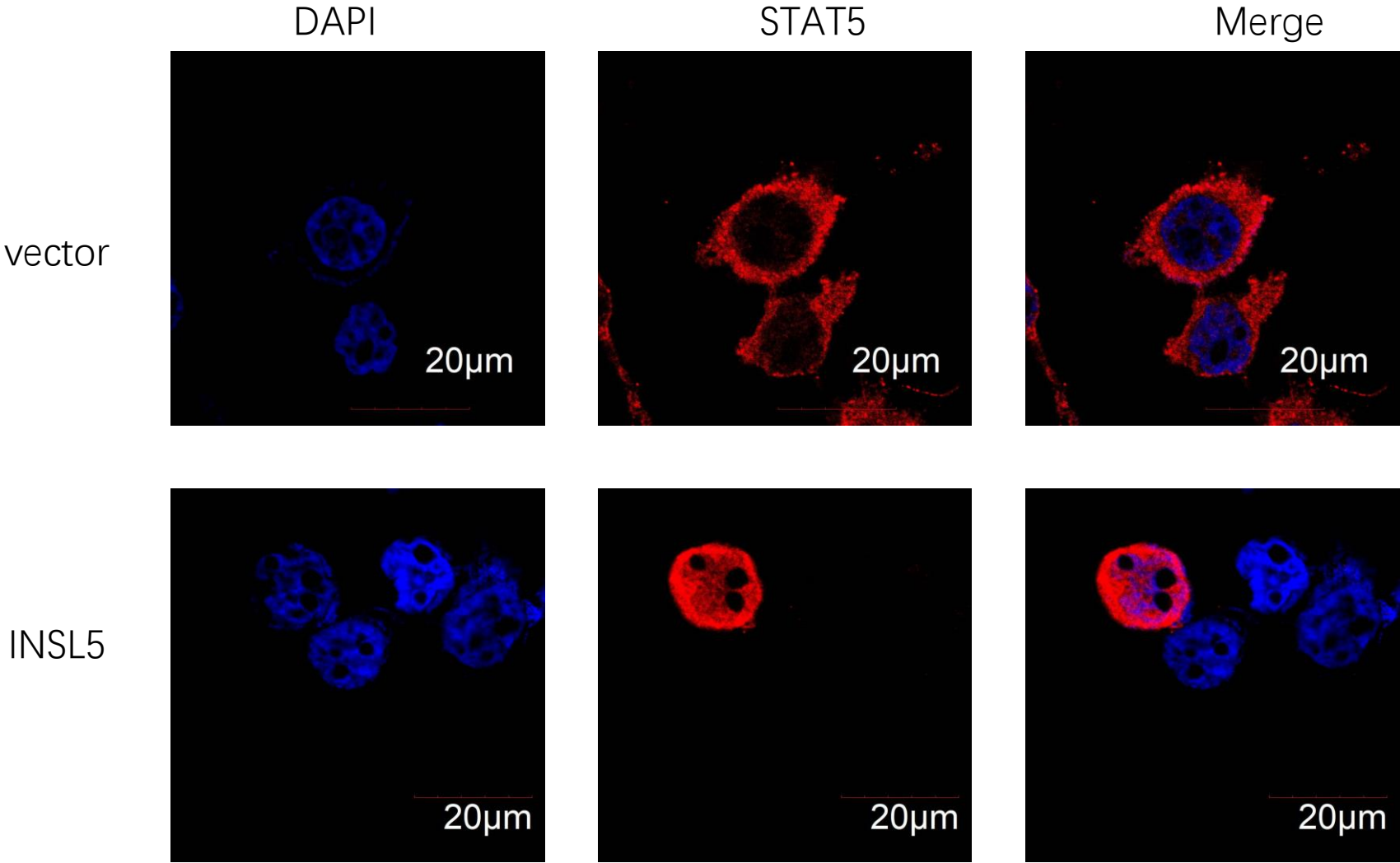

Supplement: Supplementary file 8 — Source Data for Figure 5 [file EMMM-12-e12050-s006.zip › Figure 5 Microscope SD.pdf]

Figure 6A

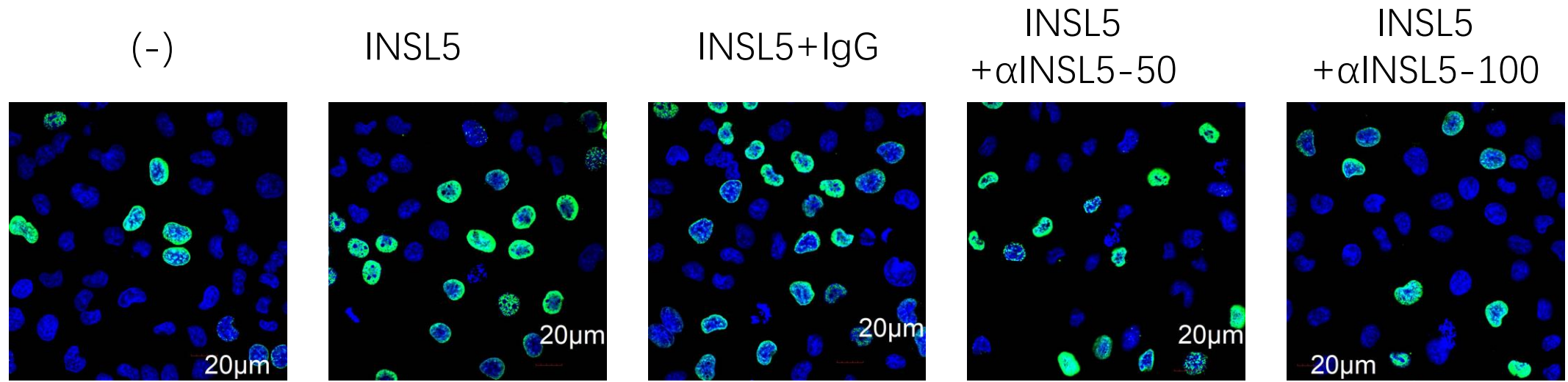

Figure 6B

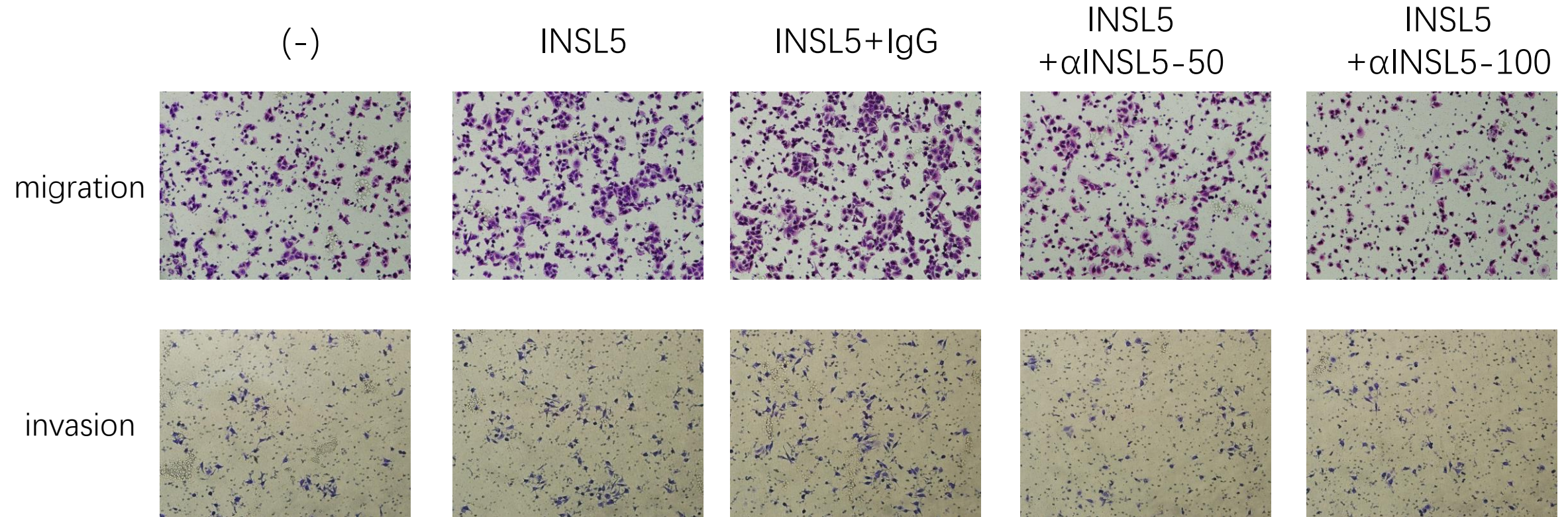

Supplement: Supplementary file 9 — Source Data for Figure 6 [file EMMM-12-e12050-s007.pdf]
