# Supplementary material for: Autocrine INSL5 promotes tumor progression and glycolysis via activation of STAT5 signaling
Source: EMBO Mol Med. 2020 Jul 12;12(9):e12050. doi: 10.15252/emmm.202012050 (PMC7507000; doi:10.15252/emmm.202012050)

Fig EV2A and EV2H

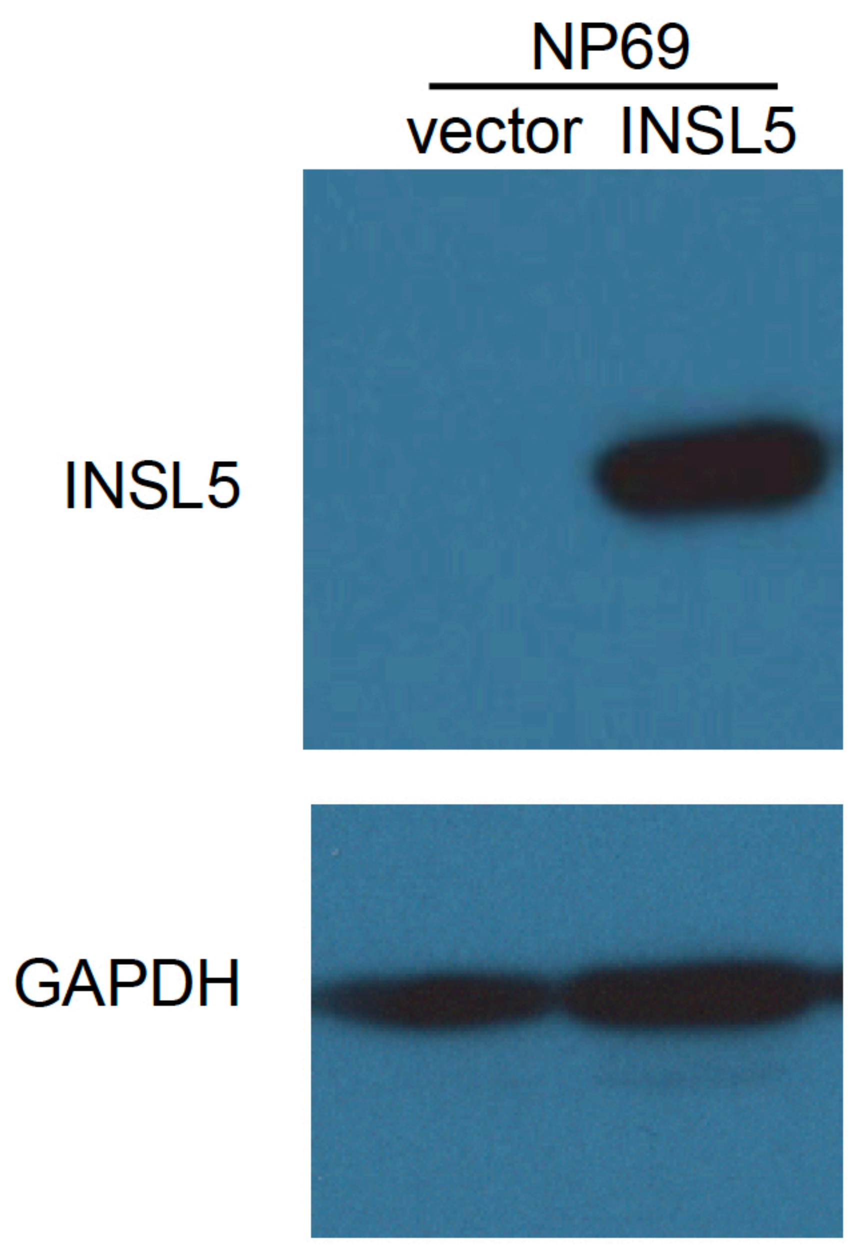

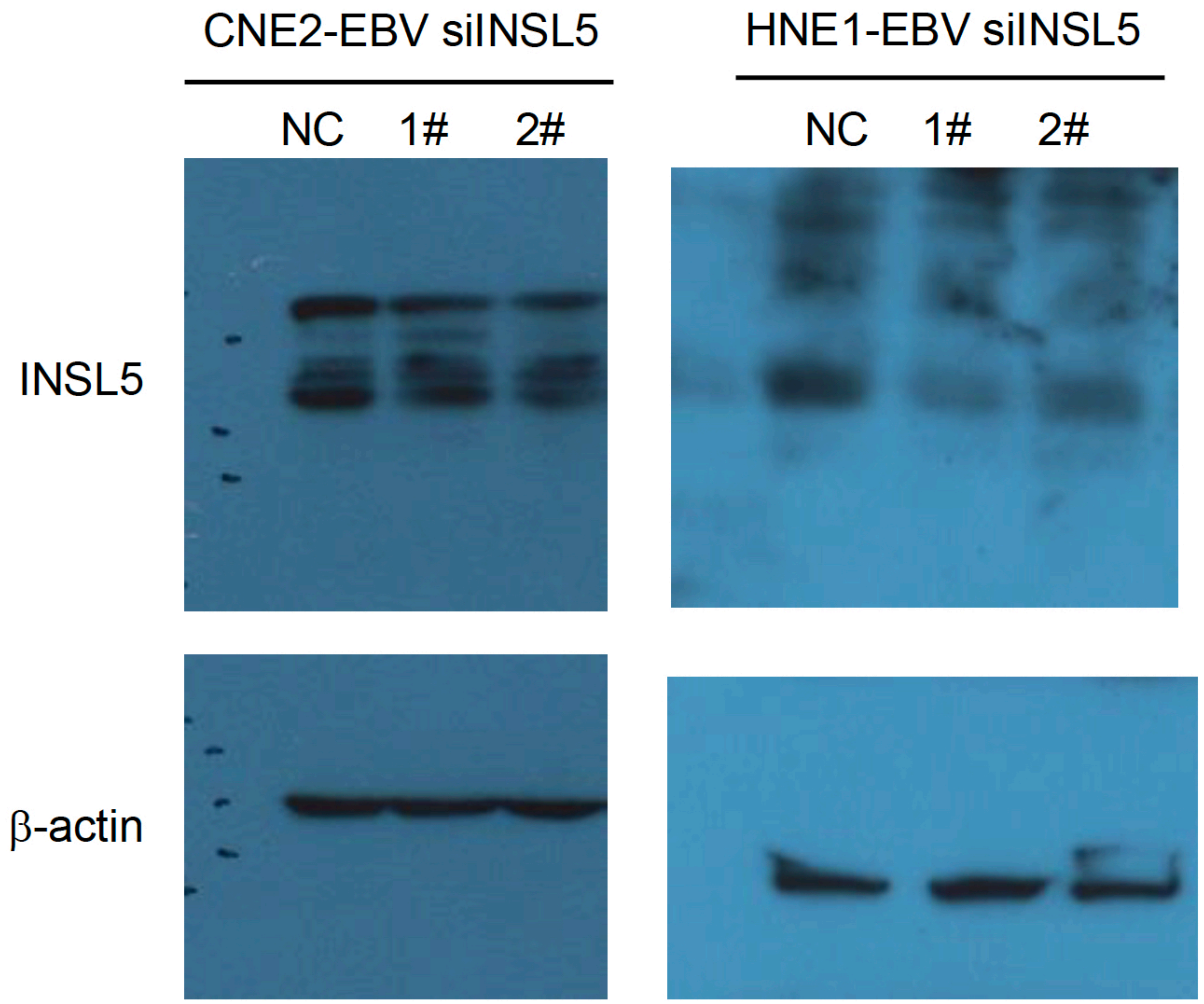

Supplement: Supplementary file 3 — Source Data for Expanded View [file EMMM-12-e12050-s008.zip › Figure EV2 Source Data.zip!/Figure EV2 western_blot_Source_Data.pdf]
