# Supplementary material for: Autocrine INSL5 promotes tumor progression and glycolysis via activation of STAT5 signaling
Source: EMBO Mol Med. 2020 Jul 12;12(9):e12050. doi: 10.15252/emmm.202012050 (PMC7507000; doi:10.15252/emmm.202012050)
Supplement: Supplementary file 3 — Source Data for Expanded View [file EMMM-12-e12050-s008.zip › Figure EV2 Source Data.zip!/Figure EV2 Microscope SD.pdf]

Figure EV2G

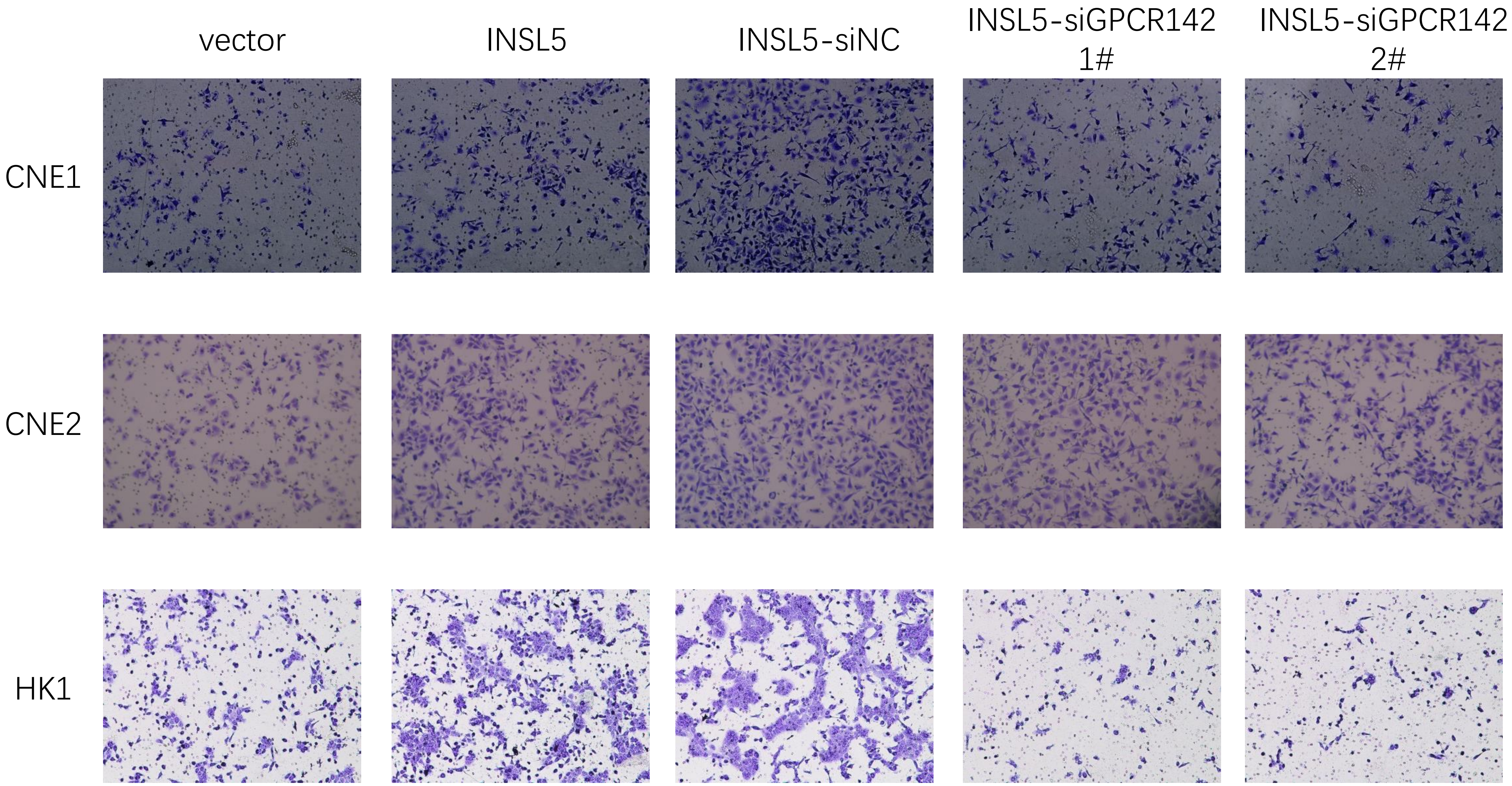
vector
INSL5
INSL5-siNC
INSL5-siGPCR142 1#
INSL5-siGPCR142 2#
CNE1
CNE2
HK1

Figure EV2I

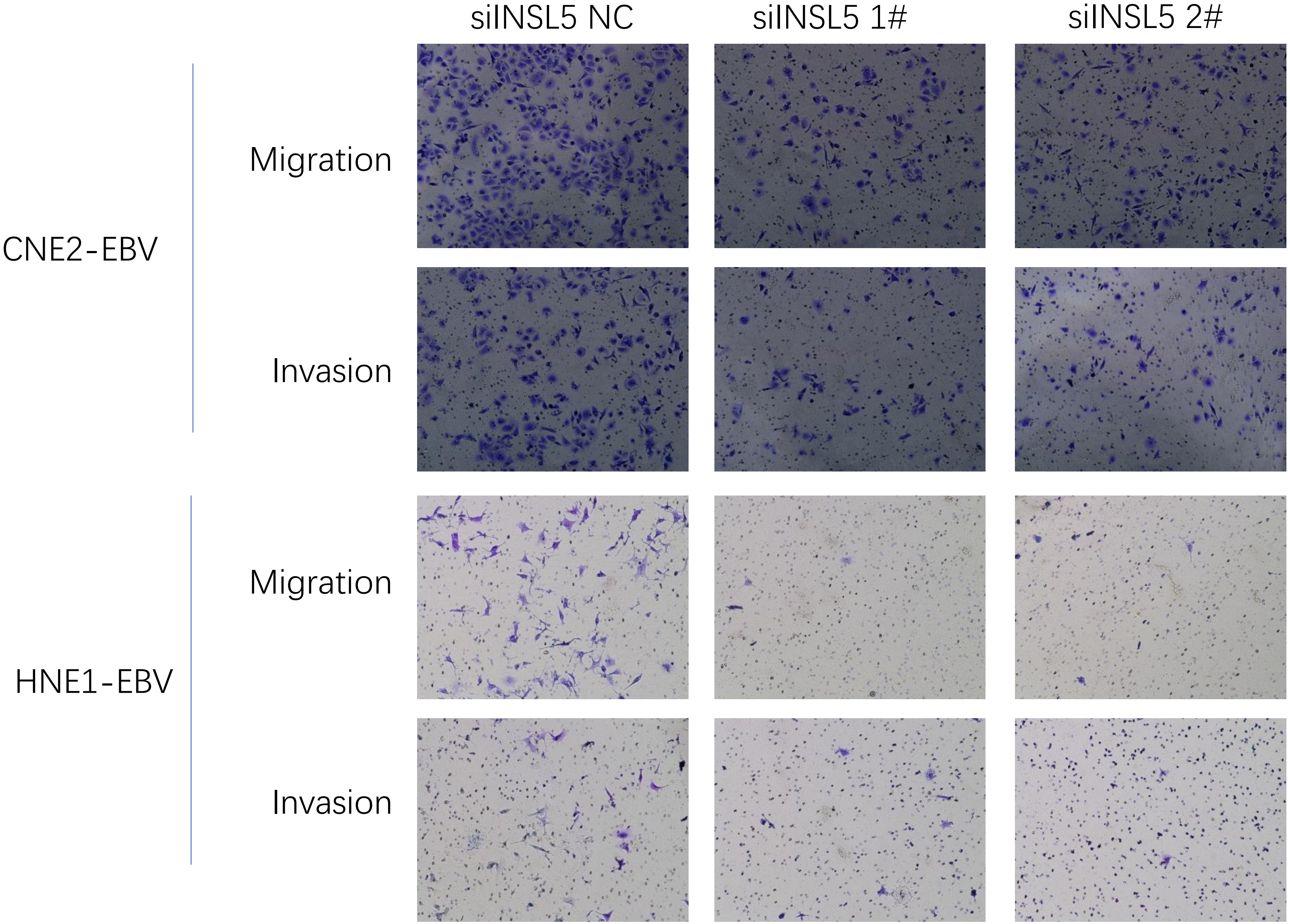
siINSL5 NC
siINSL5 1#
siINSL5 2#
CNE2-EBV
Migration
Invasion
HNE1-EBV
Migration
Invasion
